# The developmental programme for genesis of the entire kidney is recapitulated in Wilms tumour

**DOI:** 10.1371/journal.pone.0186333

**Published:** 2017-10-17

**Authors:** Ryuji Fukuzawa, Matthew R. Anaka, Ian M. Morison, Anthony E. Reeve

**Affiliations:** 1 Cancer Genetics Laboratory, Department of Biochemistry, University of Otago, Dunedin, New Zealand; 2 Department of Pathology, Tokyo Metropolitan Children's Medical Center, Fuchu, Japan; 3 Department of Pathology, University of Otago, Dunedin, New Zealand; 4 Department of Pathology, School of Medicine, International University of Health and Welfare, Narita, Japan; 5 Department of Medicine, University of Toronto, Toronto, Canada; UCL Institute of Child Health, UNITED KINGDOM

## Abstract

Wilms tumour (WT) is an embryonal tumour that recapitulates kidney development. The normal kidney is formed from two distinct embryological origins: the metanephric mesenchyme (MM) and the ureteric bud (UB). It is generally accepted that WT arises from precursor cells in the MM; however whether UB-equivalent structures participate in tumorigenesis is uncertain. To address the question of the involvement of UB, we assessed 55 Wilms tumours for the molecular features of MM and UB using gene expression profiling, immunohistochemsitry and immunofluorescence. Expression profiling primarily based on the Genitourinary Molecular Anatomy Project data identified molecular signatures of the UB and collecting duct as well as those of the proximal and distal tubules in the triphasic histology group. We performed immunolabeling for fetal kidneys and WTs. We focused on a central epithelial blastema pattern which is the characteristic of triphasic histology characterized by UB-like epithelial structures surrounded by MM and MM-derived epithelial structures, evoking the induction/aggregation phase of the developing kidney. The UB-like epithelial structures and surrounding MM and epithelial structures resembling early glomerular epithelium, proximal and distal tubules showed similar expression patterns to those of the developing kidney. These observations indicate WTs can arise from a precursor cell capable of generating the entire kidney, such as the cells of the intermediate mesoderm from which both the MM and UB are derived. Moreover, this provides an explanation for the variable histological features of mesenchymal to epithelial differentiation seen in WT.

## Introduction

The kidney develops from two distinct lineages involving a mutual inductive interaction between the metanephric mesenchyme (MM) and ureteric bud (UB) that emanates from the Wolffian duct [1]. This reciprocal inductive effect leads both lineages to develop into different types of epithelial structures [1]. The MM differentiates into glomerular epithelia, proximal tubules, and distal tubules, while the UB differentiates into the collecting ducts.

Wilms tumour (WT) is an embryonal tumour whose histology can consist of three major components (epithelial, blastemal and stromal) [2] which recapitulates the developmental phases of nephrogenesis [3], but in a disorganised manner. Some WTs additionally show myogenic and other mesodermal differentiation. In the WT that contain epithelial elements, we have noted that there are at least two different types of epithelial structures based on the morphology and WT1 immunohistochemistry [4]. One of these is a not fully differentiated epithelial structure, which is positive for WT1 immunostaining, and which might be equivalent to early glomerular epithelia of the developing kidney [4]. The other has the appearance of a fully formed epithelial structure, which is negative for WT1 immunostaining, suggesting that this type of epithelial structure is equivalent to the UB and its derivative, the collecting duct [4]. However, in spite of other supporting evidence including morphology [2, 3], lectin histochemistry [5] and ROBO1 and SLT2 immunohistochemistry [6], the presence of UB-like structures in WT remains unverified. This is possibly because a series of studies consistently demonstrated that WTs are monoclonal [7–9] which can be seen as inconsistent with the involvement of two different tissue origins. Consequently, it has been generally acknowledged, albeit with some uncertainty, that WT originates from precursor cells in the MM [10–12].

Nephrogenic rests (NR) are precursor lesions found within WT-bearing and WT predisposition syndrome-associated kidneys, which are composed of remnants of embryonal cells [2, 13]. NRs are categorized into two major types: intralobar nephrogenic rests (ILNRs) and perilobar nephrogenic rests (PLNRs) [2, 13]. ILNRs occur at deeper sites within the renal lobe and ILNR-derived tumours normally show mesenchymal (rhabdomyogenic) differentiation. PLNRs are located at the periphery of the renal lobe and PLNR-derived tumours typically have blastemal- or epithelial-predominant histology lacking myogenic differentiation. The localization of NRs and histologic elements of their associated tumours suggests the origins of precursor cells.

This study focuses on the identification of UB-like structures in WT using gene-expression profiling validated by immunofluorescence and immunohistochemistry. The results support the hypothesis that WT can arise from a precursor cell with the capacity to differentiate into both the MM and UB epithelial lineages and thereby recapitulate all phases of kidney development [14].

## Materials and methods

### WT samples

Forty-nine frozen tissues were available for RNA extraction. Total RNA was isolated from frozen samples using Tri Reagent (Molecular Research Center, OH, USA) and RNeasy purification columns (Qiagen, Hilden, Germany). These 49 New Zealand tumours (41 WT1 wild-type, 8 WT1-mutant) and additional six Japanese tumours (4 WT1 wild-type, 2 WT1-mutant) and three fetal kidneys (21, 22, and 37 weeks gestation) were prepared for formalin-fixed paraffin-embedded tissue sections. The New Zealand tumours were molecularly characterized and previously reported [4, 14, 15]. Ethics approval for this study was obtained from North Health Ethics Committee, Auckland, New Zealand and Tokyo Metropolitan Children’s Medical Center Ethics Committee. The Ethics Committees waived the need for consent from all patients on condition that the confidentiality of personal information was protected. Fetal kidneys were obtained at autopsy at Tokyo Metropolitan Children’s Medical Center after obtaining written comprehensive consent for utilizing all organ specimens for medical research and education.

### Microarray analysis

Affymetrix Human Genome U133 Plus 2.0 microarray data from Fukuzawa et al. [14] was used in this study, and 13 additional tumours were analysed on the same platform following identical protocols. Microarray data are available in the ArrayExpress database (www.ebi.ac.uk/arrayexpress) under accession number E-MTAB-3516. Microarray pre-processing was performed as described previously [14]. Differential expression analysis was performed with the ANOVA tool in Partek Genomics Suite (Partek, St. Louis, MO) with a false discovery rate cut-off of 5%.

### Real-time quantitative PCR

A subset of differentially expressed genes were validated via real-time quantitative PCR (QPCR) as described previously [14, 15]. The following taqman probes were used in this study: EYA1, Hs.00166804; SIX1, Hs00195590_m1; SIX2, Hs.00232731; GDNF, Hs00181185; RET, Hs00240887; WNT4, Hs00229142; NOTCH2, Hs.0022574; FOXD1, Hs00270117; UBE2G2, Hs.00163326. Statistical analysis of subgroups of microarray data and QPCR data were performed in GraphPad Prism (GraphPad Software, California, USA).

### Immunohistochemistry and immunofluorescence

**Fig 1:**
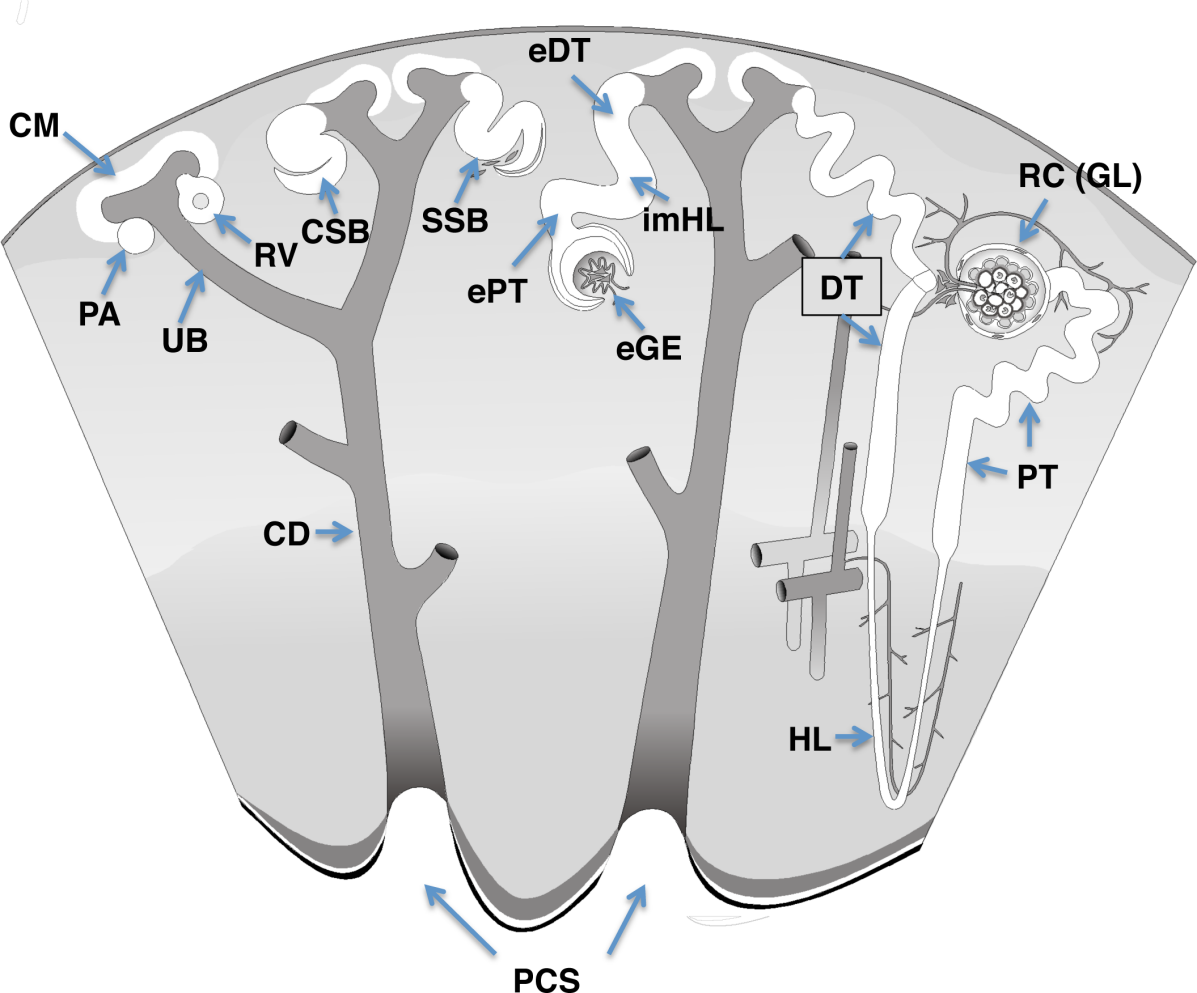
Structures of the fetal kidney. The image has been obtained from the GUDMAP database (http://www.gudmap.org/Schematics). The following abbreviations of the nomenclature of the kidney structures are used: UB, ureteric bud; CM, cap or condensing mesenchyme; PA, pretubular aggregate; RV, renal vesicle; eGE, early glomerular epithelium; CSB, comma-shaped body; SSB, S-shaped body; GL, glomerulus; RC, renal corpuscle; PT, proximal tubule; DT, distal tubule; HL, Henle Loop; CD, collecting duct; ePT, early proximal tubule; eDT, early distal tubule; imHL, immature Henle Loop; PCS, pelvi-calyceal system.

Immunohistochemistry (IHC) and immunofluorescence (IF) were performed according to standard protocols. The primary antibodies used in this study are listed in Table 1. The following secondary antibodies were used for IF: Alexa Fluor 488 goat anti-mouse IgG, Alexa Fluor 555 goat anti-rabbit IgG, and Alexa Fluor 555 goat anti-mouse IgG (Life technologies, A-11001, A-11034, and A-21422, respectively). Abbreviations of nomenclatures of fetal kidney structures are shown in Fig 1.

**Table 1:** Primary antibodies.

Gene name	Protein name	Expected Localization	Source (clonality)/Product No (clone)	Ref (animal species)
SLCO4C1	OATP-H	UB	Sigma (rp)/HPA036516	GUDMAP-A
FAM129A	FAM129A	UB/CD	abcam (rp)/ab64903	GUDMAP-A
CLMN	Protein Niban	UB/CD	abcam (rp)/ab121373	GUDMAP-A
KITLG	Kit ligand	UB/CD	LSBio (rp)/LS-B5002/69653	GUDMAP-A
MUC1	Mucin-1	UB/CD, DT	DAKO (mm)/ N1504 (E29); Thermo SCIENTIFIC (rp)/PA-5-32510; Bioss (rp)/ bs-1497R-A350	22. Leroy X et al. (Human)
CDH1	E-cadherin	UB/CD, RV	SANTA CRUZ (rp)/ sc-7870	20. Little MH and McMahon AP (Mouse)
PKHD1	Fibrocystin	UB/CD	Sigma (rp)/ HPA031229	23. Menezes LF et al. (Mouse and Human)
AQP2	AQP-2	CD	LSBio (rp)/ LS-B 1822	37. Bedford JJ et al. (Human) 28. Takasato M et al. (Human embryonic stem cell)
KRT7	Cytokeratin-7	UB/CD	Novocastra(mm), NCL-LCK7-560	24. Moll R et al. (Human)
WNT9B	Wnt-9b	UB/CD	Sigma (rp)/ HPA058361	38. Carroll TJ et al. (Mouse)
RET	c-Ret	UB	MILLIPORE (rp)/ 07–1237	41. Sainio K et al. (Rat)
SOX9	SOX-9	UB/CD, RCT, RV, SSB	R&D systems (gp)/BAF3075	GUDMAP-M
HOXD11	HOXD-11	CM, Str	LSBio (mm) /LS-C133893	30. Patterson LT et al. (Mouse)
SIX2	SIX2	CM	Protein tech (rp)/ 11562-1-AP	32. Self M et al. (Mouse)
CITED1	Cbp/p300-interacting transactivator 1	CM	Abnova (mm)/ H00004435-M03 (5H6)	11. Lovvorn HN et al. (Human)
ITGA8	Integrin alpha-8	CN, RV	ATRAS (rp)/ HPA003432	33. Muller U et al. (Mouse)
GDNF	hGDNF	CM	STANTA CRUZ (mm)/ sc-13147	41. Sainio K et al. (Rat)
WT1	Wilms tumor protein	CM, PA, RV, EGE	DAKO (mm)/ M3561 (6F-H2)	GUDMAP-M
CTNNB1	Catenin beta-1	CM, RV, UB/CD	Novocastra (mm)/ NCL-B-CAT; SANTA CRUZ (rp)/ sc-7199	4. Fukuzawa R et al. (Human)
PAX8	Pax-8	RV, EGE, UB/CD	Protein tech (rp)/ 10336-1-AP	39. Eccles M et al. (Human)
CDH6	K-cadherin	RV	LSBio (mm)/ LS-B6726/60583	GUDMAP-M
JAG1	Jagged-1	RV	Abcam (rm)/ Ab109536	GUDMAP-M
SYNPO	Synaptopondin	POD	Abcam (rp)/ ab101883	29. Mundel P et al. (Rat)
SLC3A1	NBAT	PT	Novus (rp)/ NBP1-84853	GUDMAP-A
SCNN1A	ENaCA	DT	Protein tech (rp)/ 16343-1-AP	27. Wetzel RK et al. (Rat)
KCNJ1	ATP-sensitive inward rectifier potassium channel 1	DT	ATRAS ANTIBODIES (rp)/HPA026962	GUDMAP-M
CD34	CD34	ENDO	Nichirei (mm)/NU-4A1	48. Takano K et al. (Human)
CD31	DD31	ENDO	Dako (mm)/JC70A	48. Takano K et al. (Human)
ACTA1	Actin, alpha skeletal muscle	SM	Nichirei (mm)/1A4	48. Takano K et al. (Human)

MM, metanephric mesenchyme; RV, renal vesicle; UB, ureteric bud; CD, collecting duct, PA, pre-tubular aggregates; EGE, early glomerular epithelia; SSB, S-shaped body; PT, proximal tubule; DT, distal tubule; RCT, renal crtical tubule; POD, podocyte; ENDO, endothelial cell; SM, smooth muscle; Str, stroma; GUDMAP; mm, mouse monoclonal; rp, rabbi polyclonal; rm, rabbit monoclonal; gp, goat polyclonal; A, anchor gene; M marker gene; The Genitourinary Development Molecular Anatomy Project: www.gudmap.org.; A, anchor gene; M, marker gene

### Literature search

We primarily used a database of mouse kidney development known as Genitourinary Molecular Anatomy Project (GUDMAP, www.gudmap.org) because no database for human kidney development is currently available. The GUDMAP database describes anchor genes whose expression is restricted to one temporospatial anatomical compartment and marker genes which display a regional or enriched expression pattern [16, 17]. Pubmed was also used to search for additional candidate genes for the developing kidney structures based on previous review articles [1, 18–21] and a kidney development database (http://golgi.ana.ed.ac.uk/kidhome.html).

### Identification of ureteric bud genes and ureteric bud-like structures

We undertook the following approaches to identify ureteric bud (UB)-like structures in WTs: (i) We compared the gene expression profile of triphasic tumours with that of blastemal predominant tumours. (ii) We searched the differentially expressed genes to identify candidate genes of UB and collecting duct (CD) primarily from the GUDMAP database and Pubmed (Tables 2 and 3, Fig 1). (iii) The localization of the candidate genes for UB and CD was validated in the nephrogenic zone of fetal kidneys by IHC and IF and compared with WTs. (iv) To differentiate UB-derived epithelial structures from MM-derived ones, we characterized the structures around the UB-like structures. (v) We similarly searched candidate genes for pre-tubular aggregate (PA), condensing mesenchyme (CM), renal vesicle (RV), comma-shaped body (CSB), S-shaped body (SSB), early proximal tubule (ePT) and distal tubule (eDT), immature Henle loop (imHL) listed from the differentially expressed genes (Tables 2 and 3, Fig 1). Likewise, their locations were validated by IHC and IF in the fetal kidney and compared with WTs.

**Table 2:** Differentially expressed GUDMAP anchor and marker genes in Triphasic tumours relative to blastemal tumours.

GUDMAP Category		Symbols	Probe set ID	Fold changes	P value	Another markers
UB (tip)	Anchor	SLCO4C1	222071_s_at	3.1	1.02E-05	NA
CD	Anchor	FAM129A	217966_s_at	3.2	1.81E-05	NA
			217967_s_at	4.1	1.97E-05	
		CLMN	213839_s_at	2.0	4.13E-05	
			225757_s_at	1.4	0.00351973	
	Marker	KITLG	226534_at	3.2	1.04E-05	UB (tip)
CM	Marker	EYA1	214608_s_at	-6.0	0.000760192	RI
		HOXA10	213147_at	-3.1	0.00389691	RI
		NR2F2	209121_x_at	-1.6	0.00259523	RI
PA	Marker	LHX1	206230_at	3.3	0.00322922	RV, CSB, SSB
		WT1	216953_s_at	-2.2	0.00198452	RV, CSB, SSB, GL
RV, CSB, SSB	Marker	BMP2	205289_at	3.5	0.000346047	RV, CCB, CSB
		CDH6	205532_s_at	4.7	0.00180247	RV, CSB, SSB
			214803_at	4.1	0.00478225	RV, CSB, SSB
			210601_at	1.7	0.00316506	RV, CSB, SSB
		JAG1	209098_s_at	4.3	6.73E-05	RV, CSB, CSB
			231183_s_at	3.8	5.94E-05	
			216268_s_at	4.2	8.63E-06	
			209099_x_at	3.7	8.40E-06	
		PAPSS2	203060_s_at	2.4	0.0046346	
ePT	Anchor	CRYL1	220753_s_at	1.8	0.000863014	NA
		FBP1	209696_at	2.1	0.00120846	
		SLC27A2	205769_at	2.6	5.14E-06	
		TCN2	204043_at	1.3	0.00167563	
		SLC3A1	205799_s_at	11.6	6.23E-07	
	Marker	CIDEB	221188_s_at	1.6	0.00109202	CRT, imHL
		CLIC6	227742_at	3.7	0.000634547	
		UNC5CL	231008_at	2.1	0.00117722	
		VDR	204255_s_at	2.1	0.000209724	ND
eDT	Marker	KCNJ1	210402_at	2.2	0.00141323	imHL
imHL	Marker	KCNJ1	See above	See above	See above	eDT

NA, not applicable; ND, not described; UB, ureteric bud; CD, collecting duct; CM, cap mesenchyme; RI, renal interstitium; PA, pretubular aggregate; RV, renal vesicle; CSB, comma-shaped body; SSB, S-shaped body; GL, glomerulus; CRT, cortical renal tubule; ePT, early proximal tubule; eDT early distal tubule; imHL, immature Henle loop

**Table 3:** Known kidney development genes differentially expressed in Triphasic tumours relative to blastemal tumours.

Category	Candidate Genes	Probe set ID	Fold changes	P value	Ref
UB/CD	KRT7	209016_s_at	10.3	1.5E-08	24. Moll R et al.
	PKHD1	244410_at	1.5	1.2E-05	23. Menezes LF et al.
		241694_at	6.8	1.9E-06	
	MUC1	207847_s_at	4.5	1.3E-07	22. Leroy X et al.
		213693_s_at	4.5	1.6E-07	
		211695_x_at	1.9	1.7E-05	
	CDH1	201131_s_at	8.2	0.00012	20. Little MH and MacMahon AP
	LIF	205266_at	1.9	0.00082	25. Barasch J et al.
Str	FOXD1	206307_s_at	-3.3	0.0031	36. Hatini V et al.
CM	HOXD11	214604_at	-4.6	0.00051	30. Patterson LT et al.
	SIX1	205817_at	-3.2	0.00046	31. Li CM et al.
		228347_at	-4.0	0.0018	
	SIX2	206510_at	-5.8	0.00018	32. Self M et al.
	CITED1	207144_s_at	-7.1	0.00022	11. Lovvorn HN et al.
	ITGA8	242071_x_at	-1.6	4.6E-05	33. Muller U et al.
		239092_at	-3.5	0.00023	
		214265_at	-3.5	0.00083	
		235666_at	-3.5	0.00011	
CM, PA, RV, CSB, SSB	FOXC2	214520_at	-2.9	0.0041	35. Takemoto M et al.
POD	SYNPO	202796_at	3.9	1.5E-05	29. Mundel P et al.
DT	FXYD2	205674_x_at	5.8	3.4E-06	27. Wetzel RK et al.
	SCNN1A	203453_at	6.9	1.3E-07	

UB, ureteric bud; CD, collecting duct; Str, stroma; CM, cap mesenchyme; PA, pretubular aggregate; RV, renal vesicle; CCB, comma-shaped body; SSB, S-shaped body; GL, glomerulus; PT, proximal tubule; POD, podocyte; DT distal tubule

## Results

### 1. Wilms tumour histology

**Fig 2:**
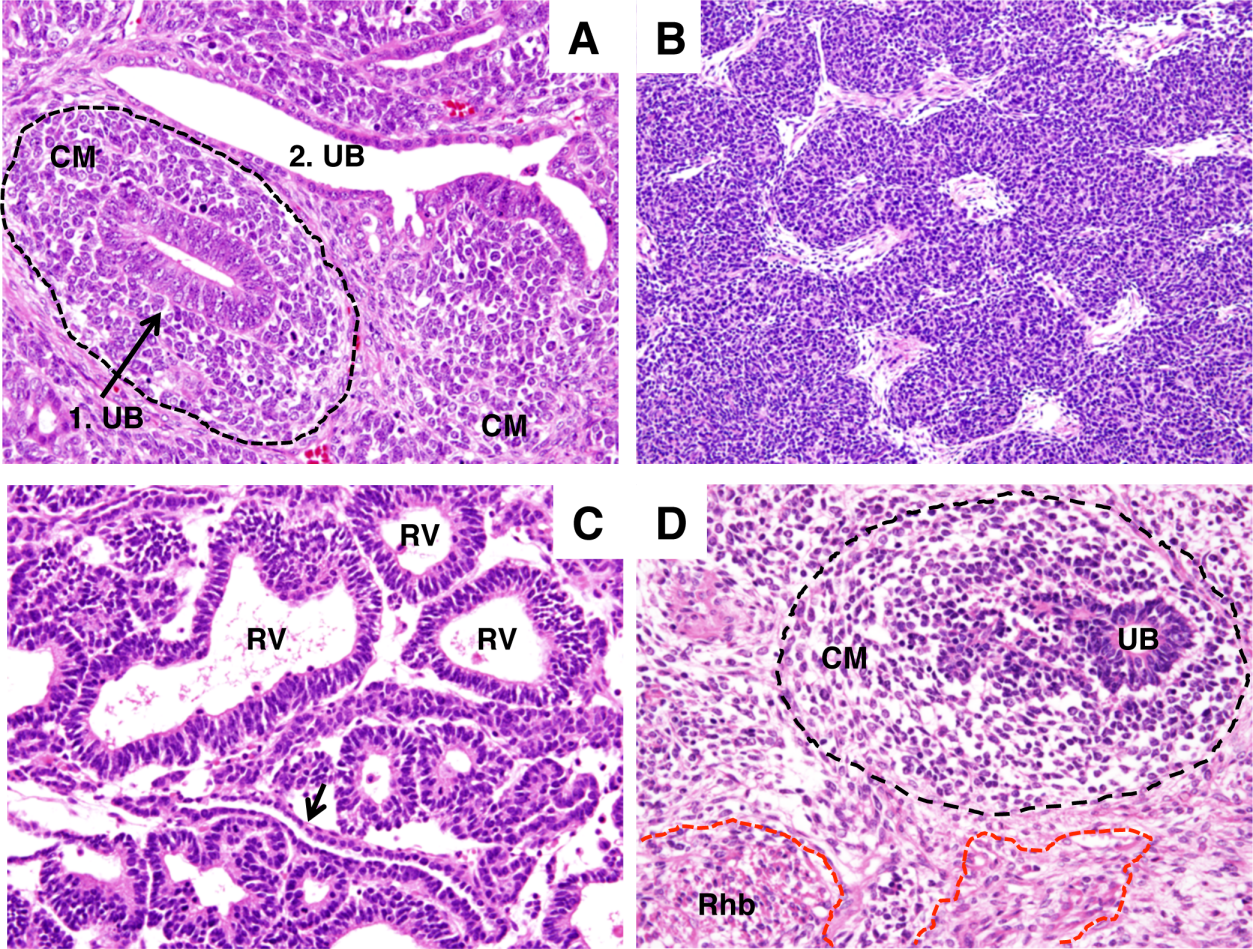
Histology of Wilms tumour. Cross section (1. UB) and longitudinal section (2. UB) of ureteric bud (UB)-like epithelial structures in a triphasic tumour. The cross section shows a central epithelial blastema pattern (indicated by arrows), and a UB-like structure surrounded by condensing mesenchyme (CM, surrounded by broken lines). (A). Wilms tumour with blastemal-predominant histology showing a typical serpentine growth pattern with few or absent UB-like structures. (B). Wilms tumour with epithelial-predominant histology showing renal vesicle-like epithelial structures and thin collecting duct-like epithelial structures (arrow). (C). Wilms tumour with a stromal-predominant histology harbouring a *WT1* mutation showing a central epithelial blastema pattern (surrounded by broken lines) with rhabdomyogenesis (Rhb, surrounded by red broken lines). (A)-(D), HE, original magnification x400.

Tumours were classified into four subtypes according to the predominant histological elements. Triphasic tumours were composed of blastemal, epithelial and stromal elements. Triphasic tumours predominantly had a central epithelial blastema pattern with aggregations of blastemal cells around the UB-like epithelial structure, forming a nodular condensed structure (Fig 2A). The central epithelial blastema pattern is commonly seen in triphasic histology (Dr Beckwith personal communication, Fig 2A) and resembles the induction/aggregation phase of the developing kidney. Consequently, this structure represents a morphological landmark for tumours containing UB-equivalent structures. The representative features of blastemal-predominant tumours, epithelial-predominant tumours, and stromal-predominant tumours are described in the Fig 2 legend (Fig 2B–2D). Of ten tumours with *WT1* mutations, nine had a stromal-predominant histology. Tumours with *WT1* mutations mimic the entire kidney development with divergent mesenchymal differentiation. UB-like structures typically found in the central epithelial blastema pattern are also a histological characteristic of WT1-mutant WTs (Fig 2D).

### 2. Ureteric bud and metanephrogenic epithelial development gene expression signatures in WT

We investigated whether WTs contained molecular features of nephrogenesis that correspond to the two distinct epithelial cell lineages: epithelial structures derived from the MM and those derived from the UB.

#### (a) GUDMAP search (Table 2)

We performed a genome-wide differential expression analysis between six tumours with triphasic histology and 16 tumours with blastemal-predominant histology, and then searched for GUDMAP anchor and marker genes in the resulting gene list. Differentially expressed anchor and marker genes for UB, CD, CM, PA, RV, CSB, SSB, ePT, imHL, and eDT identified from the GUDMAP database are listed in Table 2. There was only one UB anchor gene (*SLCO4C1*) in the GUDMAP database, which was differentially expressed in the triphasic tumours relative to the blastemal tumours. Of six anchor genes for CD, two genes (*CLMN*, *FAM129A*) were found to be differentially expressed. A marker gene for CD, *KITLG* was also identified. There were no anchor genes for CM, PA, CSB, and eDT in the GUDMAP database. The number of anchor genes for RV, CSB, ePT, and imHL in the data was one, one, 25, and one respectively, of which five anchor genes for ePT (*CRYL1*, *FBP1*, *SLC27A2*, *TCN2*, *SLC3A1*) were included. The following marker genes were differentially expressed in the triphasic tumours relative to the blastemal tumours: PA (*LHX1*), RV/CSB/SSB (*BMP2*, *CDH6*, *JAG1*, *PAPSS2*), ePT and/or imHL (*CIDEB*, *CLIC6*, *UNC5CL*, *VDR*), and early DT/imHL (*KCNJ1*). Marker genes differentially expressed in the blastemal tumours relative to triphasic tumours included CM (*EYA1*, *HOXA10*, *NR2F2*) and PA (*WT1*) genes. These data suggested that WTs contain structural elements of the whole kidney.

**Fig 3:**
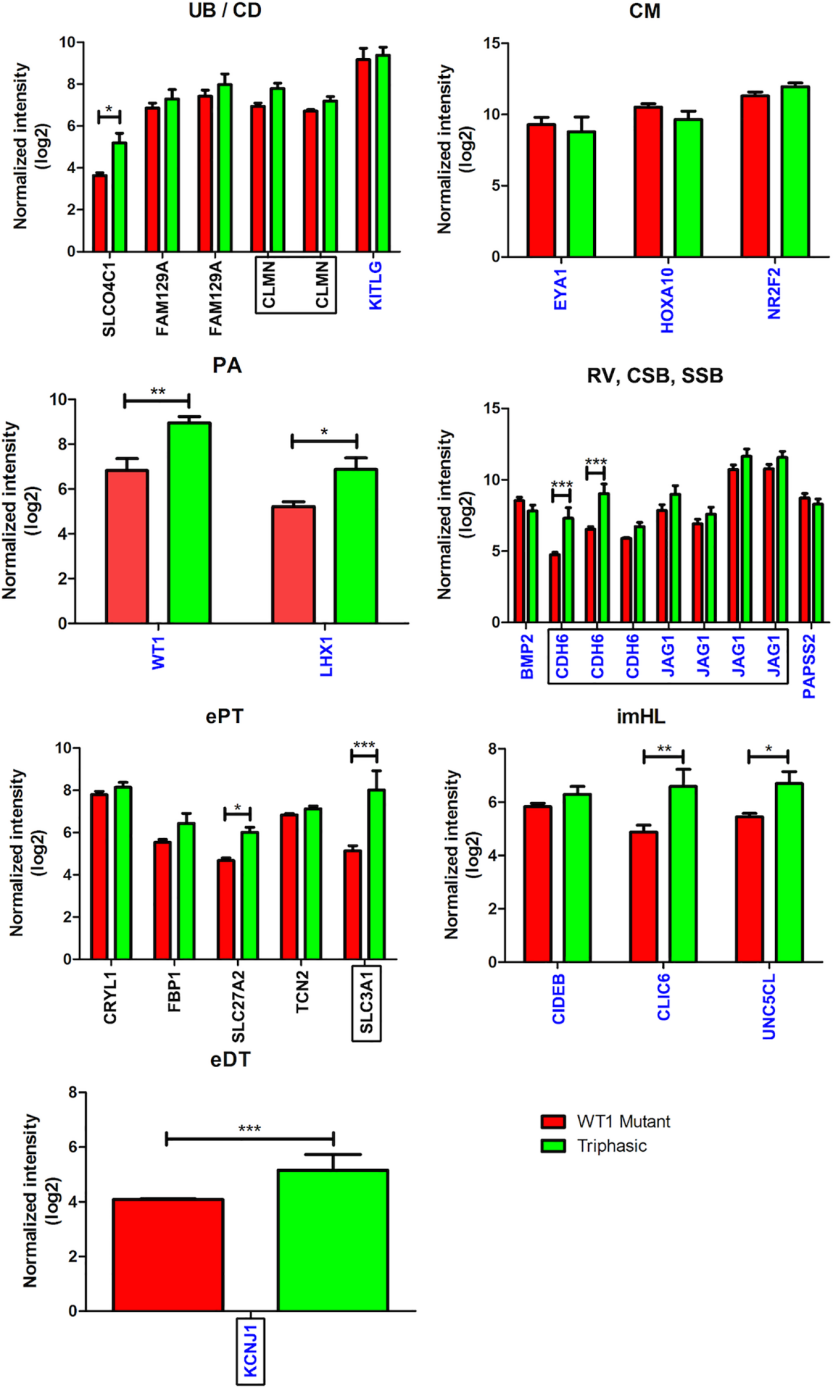
Comparison of expression levels of GUDMAP marker and anchor genes for kidney structures in WT1-mutant and triphasic tumours. Red and green bars show average log2 normalized microarray expression levels of WT1-mutant and triphasic tumours respectively. Error bars represent standard error of the mean. GUDMAP marker genes are labeled on the x-axis in blue, anchor genes in black. Some genes are shown multiple times as multiple array probe sets were identified as differentially expressed. Gene names enclosed in black squares are those with expression patterns determined by IHC/IF in this study. Genes from each structure were analysed by two-way ANOVA with Bonferroni post-tests, except for DT / KCNJ1, which was analysed by two-sided unpaired t-test. Significant interaction in the ANOVA model between tumour type and genes were identified in following structures, suggesting that genes marking these structure were consistently expressed at higher levels in triphasic tumours: RV, CSB, SSB p = 0.0003; PT p = 0.0008. * = p < 0.05, ** = p < 0.01, *** = p < 0.001.

The expression patterns of anchor and marker genes for UB/CD, PA, RV, CSB, SSB, PT, HL, and DT of WT1-mutant tumours were compared with triphasic tumours. The GUDMAP gene expression patterns for UB/CD anchor/marker genes were similar with the exception of *SLCO4C1*. Their levels of PT, RV, CSB, SSB, HL, and DT in WT1- mutant tumours appeared lower than those in triphasic tumours (Fig 3), supporting our earlier observation of the involvement of UB-like structures and disruption of metanephric epithelial development in WT1-mutant tumours [4, 15].

#### (b) Pubmed search (Table 3)

The differentially expressed genes in triphasic tumours relative to blastemal tumours included genes published as potential UB/CD markers (Table 3): *MUC1* [22], *PKHD1* [23], *KRT7* [24], *CDH1* [20], and *LIF* [25, 26]. *SCNN1A* and *FXYD2* encoding Na-K-ATPase α- and γ-subunits expressed in the distal nephron [27], a podocyte marker, *SYNPO* [28, 29] were also included.

Many genes up-regulated in blastemal-predominant tumours compared to triphasic tumours (Table 3) are known to be involved in early kidney development (stromagenic and induction/aggregation phase), including the *HOX* genes (*HOXA 10*, *11*, *HOXC 4*, *5*, *6*, *9*, *HOXD 3*, *9*, *10*, *11*) [30], the sine oculis homeobox homologues (*SIX1* [31], *SIX2* [32]), *CITED1* [11], and *ITGA8* [33, 34] (Table 3). *FOXC2*, which is the first gene expressed from the podocyte-committed region [20, 35], was highly expressed in the epithelial-predominant tumours containing abundant renal vesicle (RV)-like structures (Table 3, S4C Fig). The over-expression of *FOXD1* suggested the occurrence of stromagenesis in WTs [36].

### 3. Immunohistochemistry and immunofluorescence for molecules relevant to kidney development in fetal kidneys

**Fig 4:**
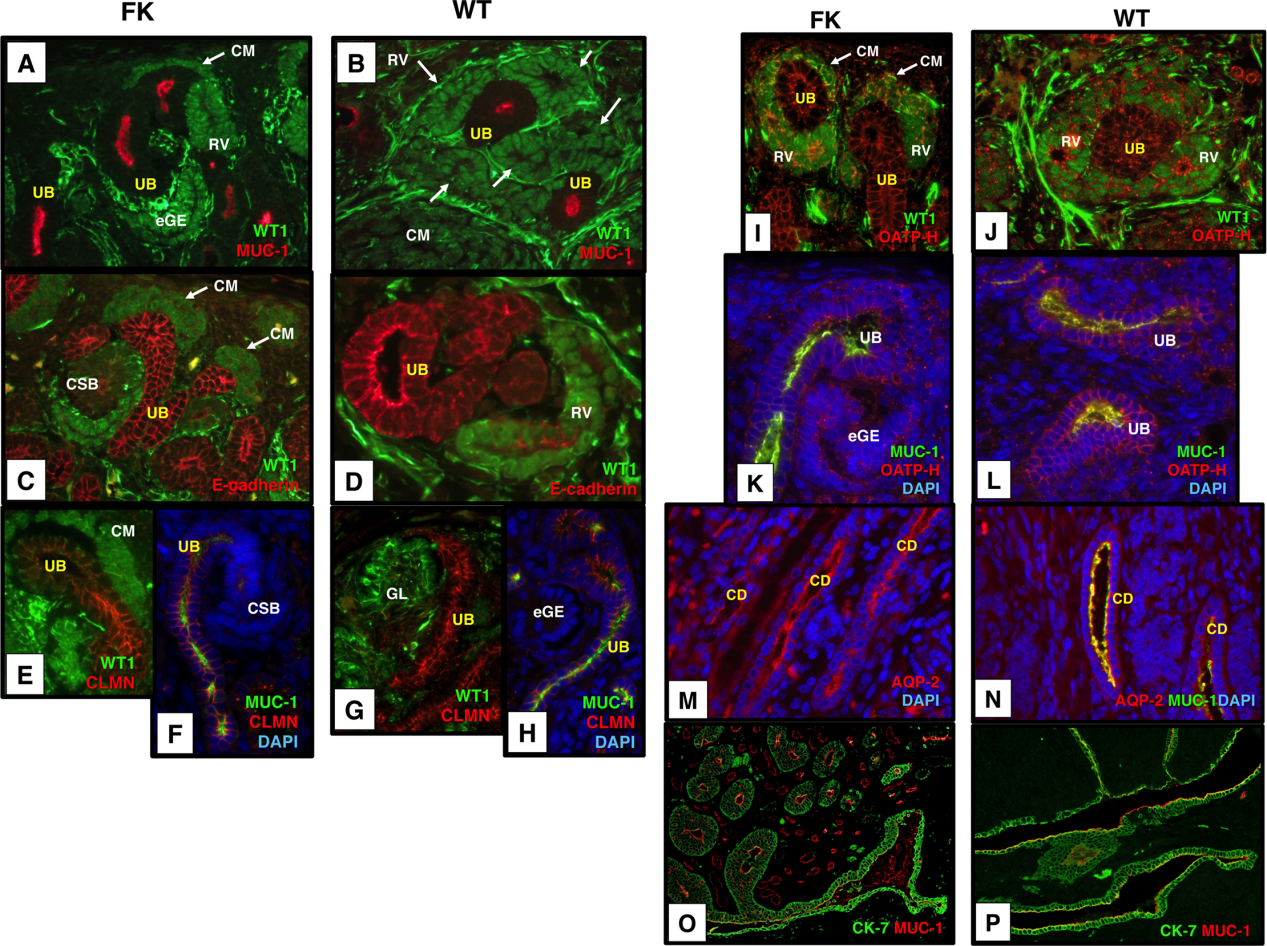
Expression of WT1, MUC1, E-cadherin, CLMN, OATP-H, AQP-2, and CK-7 in the UB/CD of FK (fetal kidneys) (A, C, E, F, I, K, M, O) and that of their equivalent structures in WT (B, D, G, H, J, L, N, P). Nuclear counterstain with 4', 6-diamidino-2-phenylindole (DAPI; F, H, K, L, M, N). (A, B) Reciprocal expression of WT1 (green) and MUC-1 (red) in FK and WT. FK (A): Absence of WT1 and expression of MUC-1 in UB, with absence of MUC-1 in condensing mesenchyme (CM) and early glomerular epithelia (eGE) where WT1 is expressed (A). WT (B): MUC-1 is expressed in lumina of UB-like structures in which WT1 expression is absent. In contrast, WT1 is expressed in nuclei of RV-like tubules (arrows) in which MUC-1 expression is absent. (C, D) Expression of WT1 (green) and E-cadherin (red) in FK and WT. FK (C): Strong membranous expression of E-cadherin in the UB where WT1 expression is absent, and weak membranous expression is seen in comma-shaped bodies (CSB, red) where WT1 (green) is weakly expressed. WT (D): E-cadherin staining is strongest in the apical membrane of the WT1-negative structures compared to that in RV-like structures. (E and F, G and H) The identical expression patterns of WT1 (green) and CLMN (red) in FK (E) and WT (G), and MUC1 (green) and CLMN (red) in FK (F) and WT (H). Membranous expression of CLMN in UB-like structures in WT (G, H) as well as in the UB in FK (E, F). Absence of WT1 and expression of MUC-1 respectively highlight the UB-like epithelial structures in WT (E, G). (I and K, J and L) Expression of the GUDMAP anchor UB gene SLCO4C1 encoding OATP-H (red), WT1 (green) and MUC-1 (green) in FK (I, K) and WT (J, L). OATP-H is localized to the cell membrane of the UB where nuclear WT1 is negative and MUC-1 is positive, whereas it is expressed in the apical membrane of RV where nuclear WT1 is positive and MUC-1 is negative in FK (I, K). The expression patterns of WT1, MUC-1 and OATP-H in FK are identical to those in WT (J, L). (M, N) Expression of a collecting duct marker, AQP-2 (red) in FK and WT. AQP-2 is expressed in the apical membrane and cytoplasm of CD in FK (M) and in those of CD-like epithelial structures expressing MUC-1 (green) in WT (N). (O, P) Expression of Cytokeratin 7 (CK7, green) and MUC-1 (red) in FK and WT. Strong CK7 expression highlighting the pelvi-calyceal system in FK (O). Likewise, CK7 is also highly expressed and co-localized with MUC-1 in large ductal structures involving stratified epithelia considered to be pelvi-calyceal differentiation in WT (P). B, D, G, H, J, L are tumors with triphasic histology group and N and P are tumours with stromal-predominant histology group. Bright signals for WT1 in glomerular tufts (the nucleus of podocytes and the cytoplasm of endothelial cells of capillaries) and the cytoplasm of some stromal cells indicate that WT1 is expressed at very high levels. (A-N), original magnification x400; (O), x100; (P), x200.

We performed IHC and IF for developing kidneys to validate the localization of the representative proteins whose genes were differentially expressed in triphasic tumours relative to blastemal tumours: Expression of GUDMAP anchor genes (*SLCO4C1*, *CLMN*, and *FAM129A*) and marker gene (*KITLG*) and putative marker proteins from the literature (*MUC-1*, *CDH1*, *PKHD1*, *KRT7*) in the UB/CD was ascertained. Additionally, proteins not listed in the profile of differentially expressed genes but known to be expressed in UB/CD, *SOX9* (GUDMAP), AQP-2 [37], and *WNT9B* [38] were assessed. MUC-1, E-cadherin, CLMN, *KRT7* encoding Cytokeratin 7 (CK7), and AQP-2 showed a differential localization pattern in the UB/CD of the nephrogenic zone of fetal kidneys (Fig 4A, 4C, 4E, 4F, 4M and 4O). Expression of *SLCO4C1* encoding OATP-H was detected in the UB, CM, and RV. While its localization in the UB was observed in the cell membrane, that in RV was predominantly in the apical membrane (Fig 4I and 4K). Therefore, we judged that its localization pattern in the UB is different from that in RV although it is not as discrete as E-cadherin and CLMN. Expression of FAM129A was low in the UB and CD (S1A Fig). The expression of KITLG, *PKHD1* encoding polycystin, SOX9, and Wnt-9b was observed in the UB/CD but they did not show differential localizations (S1C Fig and S2A, S2C and S2E Fig).

**Fig 5:**
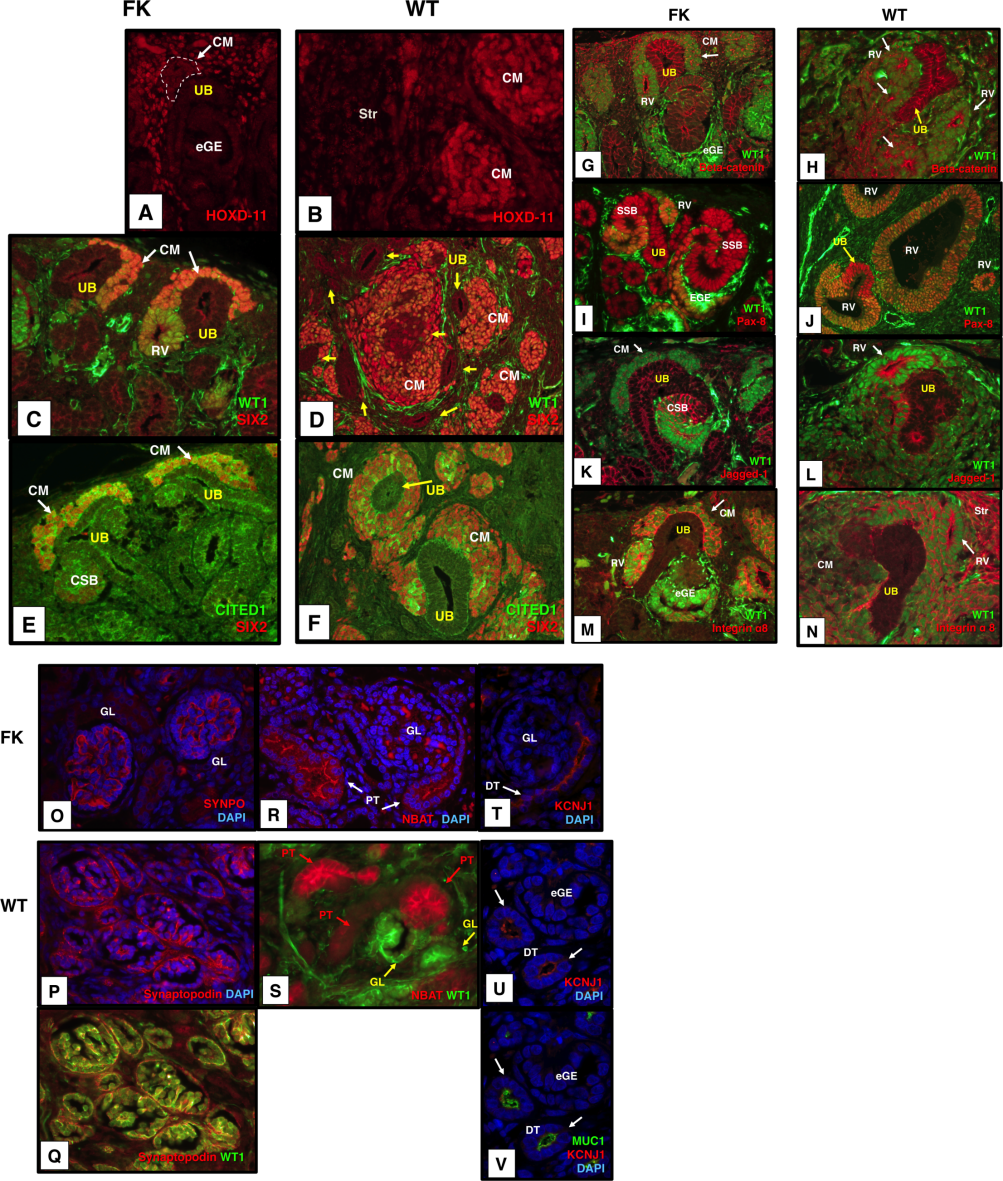
Molecular characteristics in metanephrogenic epithelial structures in FK and those in the equivalent metanephrogenic epithelial structures in WT. 1. Metanephric specification and in FK and WT. (A, B) FK (A): HOXD-11 (red) is localized in the nuclei of CM (surrounded by broken lines) as well as in those of stroma cells. WT (B): HOXD-11 (red) is expressed in the nuclei of CM-like blastemal aggregates and of stromal cells (Str). 2. Metanephric condensation in FK and WT. (C, D) FK (C): WT1 (green) and SIX2 (red) co-localized in nuclei of CM (orange nuclear signals); RVs lack expression of SIX2. WT (D): Expression of WT1 (green) and SIX2 (red) overlaps in nuclei of CM-like structures. (E, F) FK (E): Cells with nuclear SIX2 positivity (red) show nuclear and cytoplasmic expression of CITED1 (green) within the CM. WT (F): Both SIX2 (red) and CITED1 (green) positive cells are similarly present within CM-like structures. Note that the CM-like blastemal aggregates surround the WT1-negative UB-like structures and show parallel expression patterns to the CM in FK. 3. Transition from cap mesenchyme to renal vesicle in FK and their equivalent structures in WT. (G, H) Beta-catenin (red) in the cytoplasm and cell membrane of the CM and RV in FK (G) and in those of CM-like and RV-like epithelial structures in WT (H). (I, J) FK (I): WT1 and Pax-8 are co-expressed in the nuclei of RV, eGE, and lower part of S-shaped bodies (SSB, orange nuclear signals). Nuclear Pax-8 staining (red) is solely detected in UBs and maturing distal nephrons in FK. WT (J): Both WT1 and Pax-8 are expressed in RV-like epithelial structures (orange nuclear signals). Note the UB-like epithelial structure lacking nuclear WT1 (arrow). (K, L) FK (K): Jagged-1 (red) is highly expressed in the upper segment of the CSB (the proximal tubule-committed region), while its expression is weak in the apical membrane of renal lower part of the CSB. Membranous localization of Jagged-1 is noted in UBs. WT (L): Jagged-1 (red) is highly expressed in the cell membrane of RV-like structures and their derivatives where WT1 (green) is expressed. Jagged-1 is expressed in UB-equivalent epithelial structures, but its expression level is much weaker than E-cadherin and Beta-catenin. (M, N) FK (M): Integrin alpha 8 (red) is highly expressed in the cytoplasm and extracellular membrane of CM and RVs where WT1 (green) is localized in the nuclei. WT (N): Integrin alpha 8 (red) is expressed in the extracellular matrix of CM and RV-like structures, both of which express WT1. Integrin alpha 8 is also highly expressed in the stroma. Note that Beta-catenin, Jagged-1 and Integrin alpha 8 show a similar expression pattern recapitulating transition from cap mesenchyme to renal vesicle surrounding the WT1-negative UB-like structures. 4. Segmentation of GL (O), PT (R), and DT (T) in FK and their-equivalent structures (P, Q, S, U, V) in WT. (O, P, Q) FK (O): A GL marker, Synaptopodin (red) in the glomerular loop. WT (P, Q): Synaptopodin is localized to podocytes and tufts of glomeruloid structures (P) where WT1 (green) is highly expressed in WT (Q). (R, S) FK (R): A PT marker, NBAT (red) in the cytoplasm and lumen of PT. Nuclear positivity of WT1 (green) highlights the relationship between PT and GL. WT (S): NBAT (red) is positive in the epithelial structures (PT) connected to GL-like structures. (T, U, V) FK (T): A DT marker, KCNJ1 (red) in the apical membrane of DT (arrows). WT (U, V): KCNJ1 (red, indicated by arrows) in the epithelial structures adjacent to early glomeruloid epithelial structures (eGE). Co-localization of MUC-1 (green) confirmed the formation of DT (indicated by arrows). Nuclear counterstain with DAPI (O, P, T, U, V). B, D, F, H, L, N, P, S, U, V are tumours with triphasic histology group and J is a tumour from epithelial-predominant histology group. Bright signals for WT1 in glomerular tufts (the nucleus of podocytes and the cytoplasm of endothelial cells of capillaries) and the cytoplasm of some stromal cells indicate that WT1 is expressed at very high levels. (A, B, C, E, G, H, I, K, L, M, N), original magnification x400; (D, E, J), x200.

Next, the immuno-localizations of CDH6, JAG1 and beta-catenin and PAX8 [39] in RV, CSB, SSB, and SYNPO in early GL, *SLC3A1* encoding NBAT in early PT, and SCNN1A encoding ENaCA and KCNJ1 in early DT and immature HL were examined. The localizations of beta-catenin, PAX8, and JAG1 (Fig 5G, 5I and 5K), and CDH6 (S3A Fig) in the RV, CSB, SBB, SYNPO in GL (Fig 5O), NBAT in early PT (Fig 5R), and KCNJ1 (Fig 5T) and ENaCA (S3C Fig) in early DT and HL were respectively observed in the fetal kidneys.

We carried out IHC and IF for developing kidneys to examine the localization of the representative proteins whose genes were differentially expressed in blastemal tumours relative to triphasic tumours: HOXD11 was ascertained as a maker for renal stroma and MM (Fig 5A), which were localized in the uninduced mesenchyme and CM. Expression of SIX2, CITED1, and ITGA8 in CM was examined. SIX2 and CITED1were localized in CM (Fig 5C and 5E). ITGA8 was expressed in CM, RV, and eGE (Fig 5M). *WT1* is a GUDMAP marker gene for PA, which was expressed in CM, PA, RV, CSB, SBB, and glomeruli (Fig 4A, 4C, 4E, 4I and Fig 5A, 5C, 5G, 5I, 5K, 5M and 5R). These fetal kidney IHC and IF results provide a basis for comparison with WT.

### 4. Identification and validation of ureteric bud/collecting duct-equivalent epithelial structures in WTs

We examined several protein markers that distinguish UB from MM in normal developing kidneys by IHC in the series of 55 WTs. We then selected representative tumours from triphasic, epithelial-predominant, and stromal-predominant (WT1-mutant) groups for double immunofluorescence.

WT1 is highly expressed in early metanephrogenic epithelial structures in fetal kidneys, while its expression is absent in the UB of the developing kidney [4]. Meanwhile, MUC-1 is known to be expressed in the UB of the developing kidney [22, 40], which was differentially expressed in the triphasic tumours in our microarray data. We examined whether the WT1-negative and MUC-1 positive (WT1^-^/MUC-1^+^) epithelial structures (Fig 4B, S5A and S5B Fig) in WTs are equivalent to the UB of the developing kidney. Expression of either WT1 or MUC-1 was observed in two different mutually exclusive epithelial structures RV-like tubules and UB-like structures respectively in the 39 WTs with detectable WT1 protein (Fig 4B). Of 10 tumours with *WT1* mutations, WT1 protein was detectable in two tumours both of which had constitutional missense *WT1* mutations. Although the remaining eight WT1-mutant tumours lacked WT1 protein expression, MUC-1 was consistently positive in UB-like structures. Therefore, UB-equivalent structures are also present in all WT1-mutant tumours. Consistent with the reciprocal expression of WT1 and MUC-1, the remaining six blastemal-predominant tumours that all lacked WT1-negative epithelial structures were devoid of immuno-reactivity for MUC-1 in the examined sections, indicating the absence of UB-like structures.

Since E-cadherin shows a differential localization pattern in the UB compared to the early glomerular epithelium in developing kidneys (Fig 4C) [20], its expression was also examined in WTs. High levels of membranous E-cadherin expression in the WT1^-^/MUC-1^+^ epithelial structures highlighted the UB-like structures, while its expression was weak and restricted to the cell membrane in WT1^+^/MUC-1^-^ RV-like structures (Fig 4D, S5C and S5D Fig).

The other genes showing a differential expression pattern in the UB/CD of the fetal kidneys (CLMN, OATP-H, AQP-2, CK-7) were tested to confirm the presence of the UB-equivalent structures in WTs. CLMN and OATP-H were labeled in the membrane of WT1^-^/MUC-1^+^ UB-like epithelial structures in WTs (Fig 4G, 4H, 4J and 4L) as well as human fetal kidneys (Fig 4E, 4F, 4I and 4K). A collecting duct marker AQP-2 [28, 37], which co-localized with MUC-1 (Fig 4N; cf. Fig 4M), suggested that UB-like structures differentiate into collecting ducts. CK-7 was strongly expressed in the epithelial structures showing pelvicalyceal differentiation [2] in tumours with triphasic histology as well as its expression in the pelvicalyceal system of the developing kidney (Fig 4P; cf Fig 4O). The expression patterns of E-cadherin, CLMN, OATP-H, AQP-2, CK-7, MUC-1, and WT1 in the UB and/or CD-like epithelia of WTs (Fig 4B, 4D, 4G, 4H, 4J, 4L, 4N and 4P) were parallel to those in the UB of the developing kidney (Fig 4A, 4C, 4E, 4F, 4I, 4K, 4M and 4O), indicating that UB and CD-equivalent structures are present in the majority of WTs.

Although Fibrocystin, SOX-9, and Wnt-9b did not show differential localization patterns in the fetal kidney (S2A, S2C and S2E Fig), these proteins were expressed in the MUC-1^+^ UB/CD-like structures in WTs (S2B, S2D and S2F Fig). Unexpectedly, there were some inconsistencies between our results and the GUDMAP data. FAM129A and KITLG did not show differential localization patterns in UB/CD of the developing kidneys and WTs (S1A–S1D Fig). This might be due to differences in the species (mouse and human), detection methods and products (RNA and protein), and developmental stages: we used the nephrogenic zone of predominantly second trimester fetal kidneys.

GDNF and its receptor Ret provide induction signals essential for the initiation of kidney development [18, 41]. GDNF and Ret have been shown to be expressed in WTs [10, 42]. We confirmed the expression of both genes in our cohort by QPCR (S4A Fig). We ascertained co-expression of GDNF and Ret in fetal kidneys and WTs using double-immunofluorescence staining. In the fetal kidneys, while Ret was localized in the renal tubular structures including UB/CD (S2H Fig), GDNF expression was faint around and in the UB (S2G and S2H Fig). On the other hand, GDNF-expressing blastemal cells tightly aggregated around the UB-equivalent epithelial structure whereas Ret was localized mostly to the membrane of the UB-equivalent structures (S2I Fig), suggesting the potential for blastemal and ureteric epithelial cells to signal to each other. The signalling that appears to occur in this central epithelial blastema pattern (Fig 2A) corresponds to the induction/aggregation stage of the developing kidney.

### 5. Molecular and morphological characterization of the epithelial structures derived from the metanephrogenic mesenchyme

**Fig 6:**
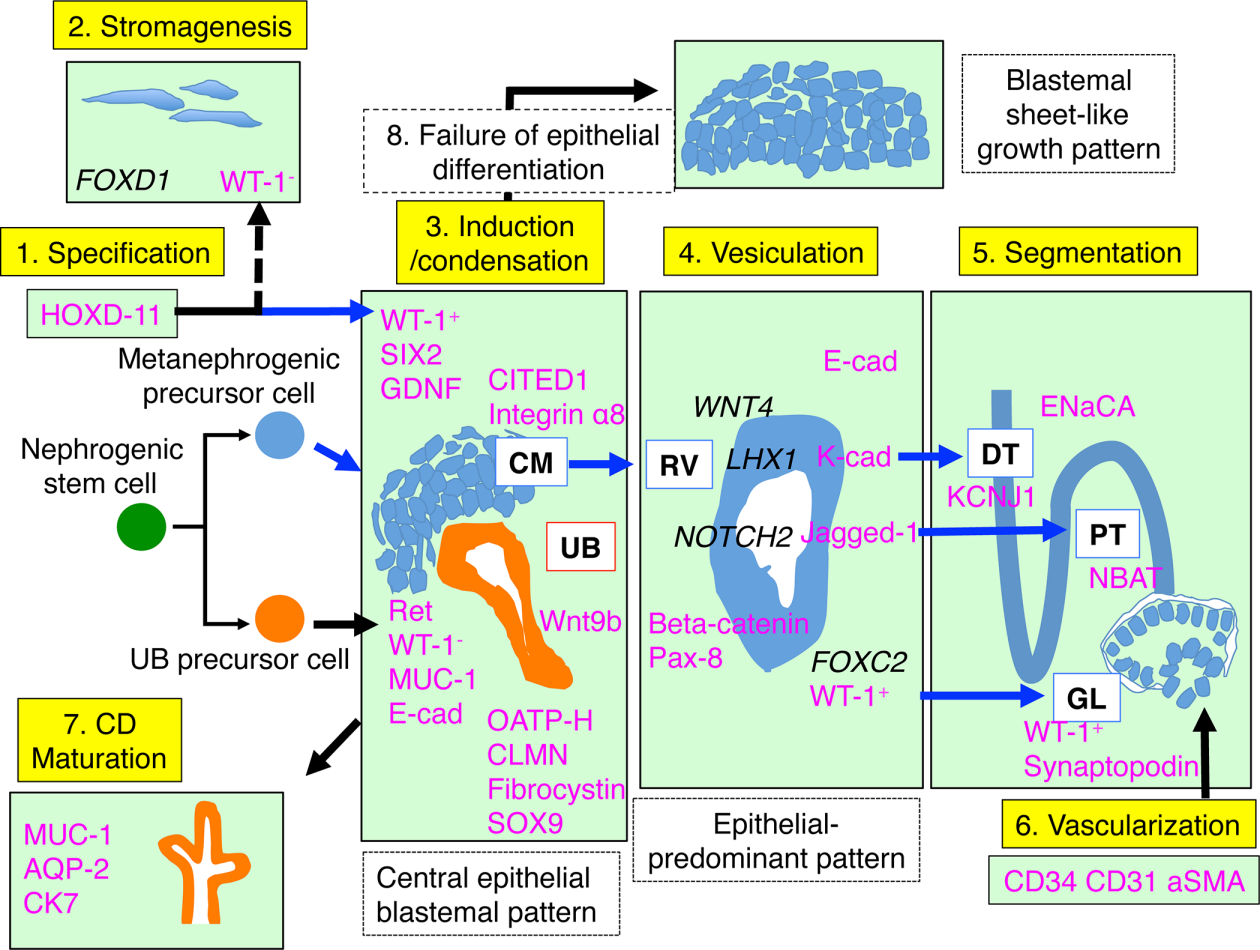
Schematic of the kidney developmental programme and histogenesis in Wilms tumour. E-cad, E-cadherin; K-cad, K-cadherin. The antibodies used as molecular markers for the metanephrogenic epithelia and UB/CD are summarized in magenta color letters. A putative nephrogenic stem cell differentiates into both metanephrogenic and UB precursor cells. Both components can interact each other and initiate the kidney developmental programme. 1, 2) Specification of the metanephric lineage and stromagenic differentiation. 3) Central epithelial blastema patterns recapitulate the induction/aggregation phase of the developing kidney, often seen in triphasic WTs. 4) Successful inductive actions allow CM to progress to RV. The RV-like epithelial structures are predominant in WTs with an epithelial predominant histology. 5) Some of the RVs might be segmented and finally differentiate into GL-, PT-, and DT-equivalent epithelial structures. 6) Vessels are incorporated into GL-equivalent structures. 7) It is conceivable that UB precursor cells can differentiate into collecting ducts- and pelvicalceal-equivalent structures. 8) If mutual induction between MM and CM is incomplete due to low numbers of UB precursor cells, blastema cells expand diffusely without driving the nephrogenic programme.

Genes that were more highly expressed in specific tumour subtypes (Tables 2 and 3) are likely to be those that are required for different components of nephrogenesis. To complete the identification of developing kidney structures, genes that were expressed highly in WTs with blastemal-predominant and triphasic histology were selected for analysis by IHC and IF (Table 1). Further support for the presence of UB-like epithelial structures in WTs was sought by confirming the expression of markers of the metanephric mesenchyme lineage in the other surrounding epithelial structures. We additionally addressed each developmental stage in order to verify the presence of the full kidney developmental programme, that can be seen in WTs, including: a. metanephric specification and stromagenesis, b. condensation of metanephric mesenchyme, c. transition from metanephric mesenchyme to renal vesicle, d. segmentation of glomerulus-, proximal tubule-, and distal tubule-equivalent structures, and vascularization). The strategy for IHC/IF based on the nephrogenic programme below (a-d) is further summarized in Fig 6.

#### (a) Metanephric specification and stromagenesis (Fig 6-1, 2)

Embryonal cells that surround the UB-equivalent epithelial structure, called blastemal cells, are considered to be equivalent to the MM. *HOXD11*, a specific marker of the metanephric region including the MM and the renal stroma [43], was expressed in the nuclei of the blastemal cells and stromal cells of WTs (Fig 5B; cf. Fig 5A). This confirms that the blastemal and stromal components of WTs are truly derived from the metanephric lineage. Expression levels of *FOXD*1, a marker for stromagenesis, were ascertained by QPCR and shown to be highest in the blastemal-predominant tumours whose histology includes few or no UB-like structures (S4B Fig). *FOXD1* is considered to be expressed in non-aggregated blastemal cells, which might share characteristics with the uninduced mesenchymal cells of the developing kidney. Therefore, expression in blastemal-predominant tumours would be expected to be higher than in triphasic or epithelial-predominant or stromal (WT1-mutant) tumours where the aggregating blastemal cells would express lower levels of *FOXD1*.

#### (b) Condensation of metanephric mesenchyme (Fig 6-3)

We next examined cap mesenchyme-equivalent structures in WTs, which are composed of densely packed MM cells closely associated with the tip of the UB in the developing kidney. Expression of WT1 overlapped with that of SIX2 (Fig 5D; cf. Fig 5C) in aggregating blastemal cells, which are considered to be equivalent to cap mesenchyme. The presence of both SIX2 and CITED1-positive cells within the cap mesenchyme of WTs (Fig 5F; cf. Fig 5E) indicates the ability to generate the entire range of epithelial structures derived from the MM, because SIX2^+^/CITED1^+^ cap mesenchyme cells in the developing kidney are nephron-committed progenitor cells capable of forming all segments of the nephron from podocyte to connecting tubule [20].

#### (c) Transition from metanephric mesenchyme to renal vesicle (Fig 6-3, 4)

RV-like structures were characterized as follows. (i) It is thought that canonical Beta-catenin WNT signaling is activated through the secretion of *WNT9B* from the UB [18]. Beta-catenin is required for renal vesicle induction, which activates early stage nephrogenic markers such as *PAX8*, *WNT4*, and *LHX1* [18, 44]. Beta-catenin and Pax-8 were expressed in the RV-like structures where WT1 was expressed (Fig 5H and 5J; cf. Fig 5G and 5I). (ii) *WNT4* also responds to *WNT9B* and prompts the expression of *LHX1* [20, 45], which is a distal nephron fate determinant that regulates the expression of another distal gene *CDH6* encoding K-cadherin [20, 46]. K-cadherin was located in the RV-like epithelial structures (S3B Fig; cf. S3A Fig). (iii) *NOTCH2* functions downstream of *LHX1* and determines a proximal nephron fate [20, 46]. Jagged-1, a NOTCH signalling receptor that is also involved in the formation of the proximal tubule [20, 46], was expressed in the RV-like epithelial structures (Fig 5L; cf. Fig 5K). Integrin alpha 8, which is required for mesenchymal to epithelial transition in developing kidneys [33], was highly expressed in the extracellular matrix of the tightly condensed MM and RV-like structures (Fig 5N; cf. Fig 5M). This indicates that the committed cap mesenchyme in WTs can also differentiate into renal vesicles, confirming that polarized epithelial structures expressing WT1 adjacent to UB-equivalent structures are equivalent to renal vesicles. The expression of *WNT4*, *FOXC2*, *LHX1*, and *NOTCH2* was confirmed by microarray and/or QPCR (S4C Fig) because effective antibodies were not available.

#### (d) Segmentation of glomerulus-, proximal tubule-, and distal tubule-equivalent structures, and vascularization (Fig 6-5, 6)

Proximal and distal tubule (PT, DT)-equivalent epithelial structures were identified in association with the presence of the glomerulus (GL)-like structure, supporting that the hypothesis that WTs recapitulate the kidney developmental programme (Fig 6): RV-equivalent epithelial formation is considered to be followed by genesis of GL-, PT- and DT-equivalent structures.

GL-like structures, which are occasionally found in WTs, are considered to be equivalent to presumptive podocytes in developing kidneys because of co-expression of podocyte markers, Synaptopodin and WT1 (Fig 5P and 5Q; cf. Fig 5O). Tubular structures equivalent to the PT were morphologically ill-defined in WTs; however *SLC3A1*, a functional marker for PT encoding Neutral and basic amino acid transport protein rBAT (NBAT) [28, 47], showed over-expression in our microarray data (Table 2). The localization of NBAT in the tubules connected to GL-equivalent structures confirmed the presence of PT-equivalent structures (Fig 5S; cf. Fig 5R). Both KCNJ1 and ENaCA co-localized with MUC-1 in well-differentiated thin tubular structures, confirming the formation of DT-equivalent structures [27] (Fig 5U, V; cf. Fig 5T, S3D and S3E Fig; cf. S3C Fig).

Incorporation of vessels into the GL-equivalent structure, which is considered to correspond to the “vascularization phase” of WTs and developing kidneys was validated by endothelial markers (CD31, CD34) and alpha smooth muscle actin (SMA) [48](S6B, S6D and S6F Fig; cf. S6A, S6C and S6E Fig).

## Discussion

We have verified that UB-like structures in WT have the protein expression characteristics of UB-derived structures in normal fetal kidney. The lack of established specific markers for UB in humans and occasional overlap of putative UB markers with other epithelial structures such as the distal nephron that occur during development represent a limitation for the pathological identification of UB-like structures in WTs. Here we utilized the histological central epithelial blastema pattern as a clue for distinguishing UB-like from RV-like structures, and furthermore used a validation of GUDMAP data on human fetal kidney tissues to better characterize lineage specific markers. Then, by examining the staining pattern and localization of proteins of putative UB markers and by differentiating them from surrounding epithelial structures, we concluded UB-like structures are involved in WTs.

The possibility that these UB-like structures are normal ureteric buds embedded in the WT can be excluded as ureteric buds do not exist in the normal juvenile kidney tissues resected with WT, having disappeared at around 34–36 weeks of gestation. Therefore, normal ureteric buds cannot be involved in Wilms tumour tissues. Ureteric buds can be seen in nephrogenic rests which are pre-neoplastic lesions, most of which have genetic and/or epigenetic alterations [49–51]. In addition, UB-like structures are morphologically obviously neoplastic because they have nuclear and structural atypia.

Through detection of this UB-equivalent component, we therefore show that the majority of WTs possess the ability to differentiate into the two lineages of nephrogenesis: the metanephric mesenchyme (MM) and the ureteric bud (UB). The MM and the Wolffian duct-derived UB are two independent tissue compartments that arise from the precursor embryonic tissue, the intermediate mesoderm [1]. All seven tumours carrying constitutional *WT1* mutations had the protein-expression characteristics of UB-derived structures, supporting the hypothesis that stem cells with the initiating mutations develop into both tumorigenic MM and UB cells. Our observations, together with the well established understanding of the ontogenesis of the fetal kidney [1], strongly support an early stem cell origin of WT. Since, as expected, WTs are monoclonal [7–9], the presence of the two epithelial structures is best explained by an intermediate mesoderm-like cancer stem cell (nephrogenic stem cell) that has the potential to differentiate into both structures. In addition, it is conceivable that the interplay between the two lineages in WT drives the nephrogenic developmental programme.

**Fig 7:**
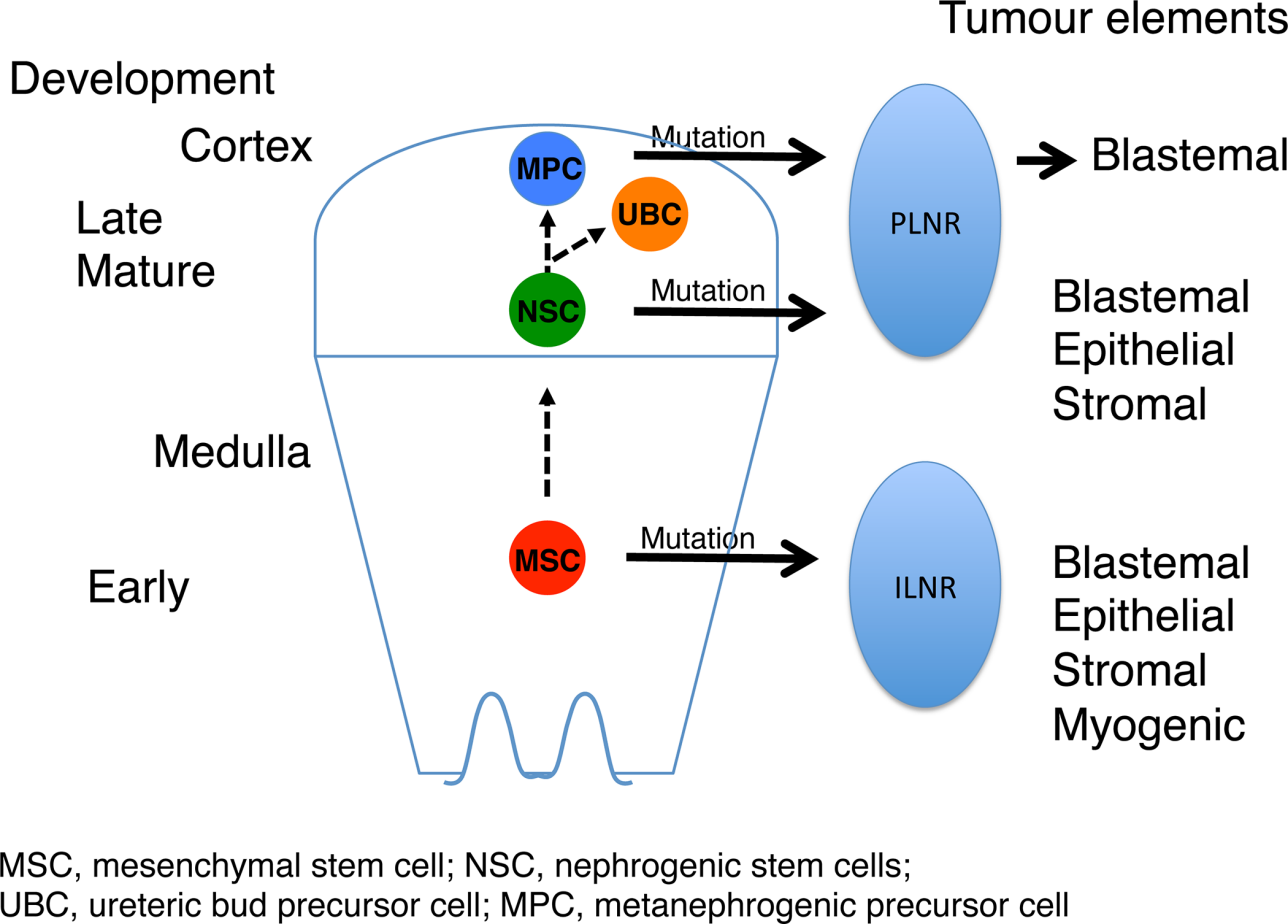
Stem cell origin hypothesis of Wilms tumour. ILNR occurs early in kidney development; thus it may predominantly contain pluripotent precursor cells that can differentiate into both mesenchymal and nephrogenic lineages. The tumours arising from this precursor cell type (mesenchymal stem cell) show divergent mesenchymal differentiation. PLNR occurs late in the nephrogenesis; therefore it may include more mature precursor cells that exhibit limited differentiation into both metanephric and ureteric precursor cells. Tumours originating from this precursor cell type (nephrogenic stem cell) show triphasic, epithelial-predominant, or blastemal-predominant histology that lacks myogenic elements. Tumours with a purely blastemal or an epithelial component might have arisen from metanephrogenic precursor cells that differentiate into the metanephrogenic lineage only.

It has been reported that cells within the MM can differentiate into both metanephrogenic and ureteric epithelia *in vitro* [52], suggesting the persistence of pluripotent renal stem cells (nephrogenic stem cells) within the MM tissue of the developing kidney. We and other groups have shown that ILNR-associated tumours have molecular features that are characterized by genes expressed in the paraxial mesoderm [14, 15] and intermediate mesoderm [53]. The location of NRs within the kidney and the histological features (presence or absence of myogenesis) of the associated tumours indicate ILNRs occur early in kidney development, while PLNRs are formed late in nephrogenesis [2, 13] (Fig 7). Therefore, ILNRs predominantly contain precursor cells committed to nephrogenic and divergent mesenchymal lineages, while PLNR is predominantly composed of precursor cells with more restricted potential committed to the nephrogenic lineage.

The sequential nature of nephrogenic rest-WT development, and our past [14, 15] and current expression profiling data of WTs indicate that WT can arise from precursor cells of different maturational states (Fig 7). A large expression-profiling study also demonstrated that ILNR-derived tumours had two different classes of molecular characteristics and different proportions of myogenic elements [53], suggesting the existence of precursor cells of different maturational stages (developmental potential) within NRs. Interestingly, a group of tumours were not associated with NRs and showed post-induction nephron signatures. We speculate that those tumours might be derived from a metanephric precursor cell whose differentiation is limited to metanephrogenic epithelia (Fig 7).

The characterization of gene and protein expression of metanephric and ureteric epithelia enabled us to interpret the histogenesis of WT. It is conceivable that as the MM and UB-equivalent epithelial cells interact with each other, the efficiency of interactions between the components and/or the number of the ureteric epithelium could determine the shape and proportion of the blastemal component (Fig 6). Appropriate interactions between the MM and UB lead to metanephrogenic epithelial development, which typically forms RV-equivalent epithelium that sometimes gives rise to GL-, PT-, and DT-equivalent structures sequentially (Fig 6) according to the kidney developmental programme. Such metanephrogenic epithelial development cannot proceed if *WT1* is mutated as we have shown in WT1-mutant WTs [4, 15]. WTs with an epithelial-predominant histology represent the stage of metanephric epithelial development in which RV-like epithelial structures are prominent (Figs 2C and 6). If the ureteric bud component is absent or minor, a blastemal-predominant histology would result (Figs 2B and 6). A subset of blastemal-predominant tumours might be purely of metanephrogenic precursor cell origin (Fig 7), as those tumours lack UB-equivalent structures. Such tumours might have a restricted range of differentiation and only show metanephric blastema and its derived epithelia.

Recent studies on cancer stem cells (CSCs) from WT xenograft models have proposed a nephrogenic/stromal developmental lineage origin for WT [54, 55]. A series of studies on a population of cells marked by NCAM1 and ALDH1 with CSC characteristics identified in 5 lines of blastemal-predominant phenotype WT xenografts derived from two triphasic WTs suggested that self-renewing human WT CSCs can constantly evoke blastemal and differentiated renal tubular elements that exist in the blastemal element of WT [54]. Furthermore, a study demonstrated that this ALDH1+ WT CSC population expressed markers suggestive of commitment to epithelial lineages, but could also give rise to cells expressing mesenchymal/stromal lineage markers, suggesting the ability to dedifferentiate in vitro [55]. These data do not directly support our results identifying the presence of UB components. However while the models characterize a type of WT histology, they are not able to fully explain the characteristic features of classical WTs containing ectopic mesenchymal elements such as tumorigenic rhabdomyoblasts. Dedifferentiation of CSCs is one explanation for stromal and divergent mesenchymal differentiation. However the two precursor lesions of WTs, in which CSCs are considered to reside, might have a clue to understand the inconsistency between the currently described experimental CSC models and the observed spectrum of WT histology. PLNR is composed predominantly of an embryonal type of cells (blastemal cells). Tumours arising from PLNR are predominantly blastemal with occasional epithelial components and usually lack ectopic mesenchymal elements, which closely resemble the aggregation phase [condensing blastema (mesenchyme)] of the developing kidney. The CSCs within PLNR-derived WTs have restricted potential committed to the nephrogenic lineage, which is consistent with the published CSC models derived from blastemal-predominant WT xenografts. Meanwhile, ILNR are composed of spindle cells considered to be equivalent to uninduced mesenchymal cells, which correspond to the stromagenic phase (non-aggregated nephrogenic mesenchyme) of the developing kidney. Tumours derived from ILNR are composed of three components (blastemal, epithelial, and stromal), of which stromal components are abundant including ectopic mesenchymal elements, and often show the entire range of kidney epithelial structures. The term “stroma” in WT does not mean connective tissue stroma [3](J.B.Beckwith personal communication). Thus, the stromal component is not derived from the blastemal component, but the blastemal component is derived from the stromal component [3](J.B.Beckwith personal communication). Therefore, it is conceivable that the CSCs within ILNR-derived WTs are more primitive state than those within PLNR-derived WTs. The published CSCs were obtained from human WT blastemal components, which would be expected to contain committed renal progenitors. If CSCs could be obtained from ILNR-derived WTs and their differentiation potential assayed, our question of the involvement of UB and the origin of ectopic mesenchymal elements might be clarified. Supporting our model, a transgenic mouse model of WT based on induction of Lin28 into nephrogenic lineage (wt1/six2) cells of the intermediate mesoderm generated triphasic WT, but no tumours were formed with induction of the Lin28 transgene in more differentiated cell types such as the cap mesenchyme, ureteric bud, or stroma [56]. The histology of the mouse WT in that study further shows a triphasic histology containing a central epithelial blastema pattern, supporting the involvement of a ureteric bud equivalent epithelial component in WT derived from the intermediate mesoderm cells which give rise to the entire kidney [56].We suggest that WTs arise from different maturational stages of CSCs (Fig 7), determining their histopathological features (Fig 6).

From our expression-profiling studies, we reaffirmed the molecular definition of embryonal tumours: embryonal tumours follow the developmental programme of the organ from which they arise and a cellular lineage-associated oncogenic programme. However, malignant embryonal tumours show a failure of complete differentiation of their original organs. Similarly, it is anticipated that cells with pluripotent properties such as Embryonic Stem cells and inducible Pluripotent Stem cells could become germ cell tumours and embryonal tumours according to their developmental stages, if an oncogenic programme is activated.

## Supporting information

S1 Fig IHC for FAM129A (A, B) and KITLG (C, D) in FK and WT.
**(**A, B) Expression of FAM129A highlights vessels in FK (A) and WT (B).
(C, D) FK (C): KITLG is expressed not only in the UB but also in maturing and mature renal tubules in the cortex. WT (D): Expression of KITLG is weak in the UB/CD-like structures (arrows). Original magnification, A-D, x400. Nuclear counterstain with 3, 3’-diaminobenzidine (DAB)
(TIF)

S2 Fig Expression of Fibrocystin (A, B), SOX9 (C, D), Wnt-9b (E, F), GDNF (G, H, I) and Ret (H, I) in FK and WT. Fibrocystin, SOX-9, Wnt-9b, and Ret did not show differential expression in fetal kidney but were expressed in the UB-like structures.
(A, B) FK (A): Expression of Fibrocystin (red) in the apical membrane of a ureteric bud overlapping MUC-1expression (green). WT (B): Photomicrograph showing a central epithelial blastema pattern in which Fibrocystin (red) and MUC-1 (green) are localized to the apical membrane and cytoplasm of the UB-like structure.
(C, D) Double IF for SOX-9 (red) and WT1 (green) in FK and WT.
FK (C): SOX9 is expressed in the UB tip. WT (D): SOX-9 is expressed in a WT1-negative epithelium and its expression is absent in a WT1-positive epithelium (indicated by an arrow).
(E, F) Double IF for Wnt-9b (red) and MUC-1 (green) in FK and WT.
FK (E): Wnt-9b in the apical membrane, cell membrane, and cytoplasm of UBs. WT (F) shows an identical expression pattern in the MUC1-positive UB-like structures.
(G, H, I) Expression of GDNF (green) and Ret (orange) in FK and WT.
FK (G, H): GDNF is detected in and around the UB using DAB as a substrate (G). Expression of GDNF is nearly absent while that Ret is positive in the apical membrane of UB (H). Ret is also scattered in CM. WT (I): GDNF (green) and Ret (orange) are co-expressed in UB-equivalent structures and the surrounding condensing blastemal cells (CM).
Original magnification, A-F, H, x400; G, x600 I, x200.
(TIF)

S3 Fig Expression of DT-related proteins [K-cadherin (A, B), and ENaCA (C, D, E)] in FK and WT.
(A, B) FK (A): K-cadherin in the cell membrane of the CSB, SSB, and eGE. Its expression is also observed in ePTs and eDTs in the cortex. WT (B): Membranous and/or cytoplasmic K-cadherin expression in epithelial structures connected or adjacent to the GL-like structures (arrows). This indicates K-cadherin is involving in the formation of PT and DT.
(C, D and E) FK (C): A distal tubule marker, ENaCA (red) in DT. WT (D, E): ENaCA (red) in the epithelial structures adjacent to eGE. Co-localization of MUC-1 (green) confirmed the formation of the DT (arrows).
Original magnification, A-E, x400. Nuclear counterstain with DAB (A, B).
(TIF)

S4 Fig Expression of *FOXC2*, *LHX1*, *WNT4*, and *NOTCH2* in WTs. A) Expression of *GDNF* and *cRET* in WTs. GDNF and its receptor *cRET* are expressed in all types of WTs, with higher expression of *GDNF* seen in WT1-mutant and blastemal-predominant tumours. Data shown are log2-transformed QPCR expression levels normalized to *UBE2G2*.
B) Expression of *FOXD1* in WTs. Expression of *FOXD1* is highest in blastemal-predominant tumours whose histology consists of non-aggregated blastemal cells with few or no UB-like structures. Data shown are log2-transformed QPCR expression levels normalized to *UBE2G2*.
C) Expression of *FOXC2*, *LHX1*, *WNT4*, and *NOTCH2* in WTs. *FOXC2* and *LHX1* are highly expressed in tumours with epithelial-predominant histology, which are predicted to be expressed in RV-equivalent structures. *NOTCH2* was expressed in all histological-subtypes, with the highest expression in epithelial-predominant tumours. The graph for *WNT4* shows log2-transformed QPCR expression levels normalized to *UBE2G2*. The second graph shows microarray log2-transformed normalized expression levels.
For all graphs expression levels were compared by two-way ANOVA with Bonferroni post-tests, * = p < 0.05, ** = p < 0.01, *** = p < 0.001.
(TIF)

S5 Fig Opposite expression of MUC1 and WT1 in two different epithelial structures in a Wilms tumour in the triphasic group. Consecutive sections show that WT1 is expressed in RV-like structures (A, surrounded by red circles) in which MUC1 expression is absent (B, indicated by arrows), in contrast, MUC1 is expressed in the UB-like structures (B) in which WT1 expression is lost (A, indicated by arrows). WT1 is also expressed in blastemal cells around the UB-like structures.
WT1 (A), MUC1 (B), original magnifications: x400 (A, B). Nuclear counterstain with DAB.
Nuclear immuno-positivity for WT1 highlights a cluster of RV-like cells (surrounded by a red broken circle) in a triphasic WT (C, D), while nuclear WT1 expression is absent in UB-like structures (C). E-cadherin immunostaining reveals its presence of UB-like structures surrounded by condensing mesenchyme (CM) (D).
WT1 (C), WT1 and E-cad (D), original magnifications: x200 (C, D).
(TIF)

S6 Fig The vasculization phase [CD34 (A, B), CD31 (C, D), alpha SMA (E, F)] in FK and WT.
FK: CD34 (A), CD31 (C), and alpha SMA (D) revealing vasculogenesis in eGE and G (arrows). WT: CD34 (B), CD31 (D), and alpha SMA (F), An area of the formation of GL-like structures showing vasculogenesis.
Original magnification, A-F, x400. Nuclear counterstain with DAB.
(TIF)
