# Supplementary figures and images for: The developmental programme for genesis of the entire kidney is recapitulated in Wilms tumour
Source: PLoS One. 2017 Oct 17;12(10):e0186333. doi: 10.1371/journal.pone.0186333 (PMC5645110; doi:10.1371/journal.pone.0186333)

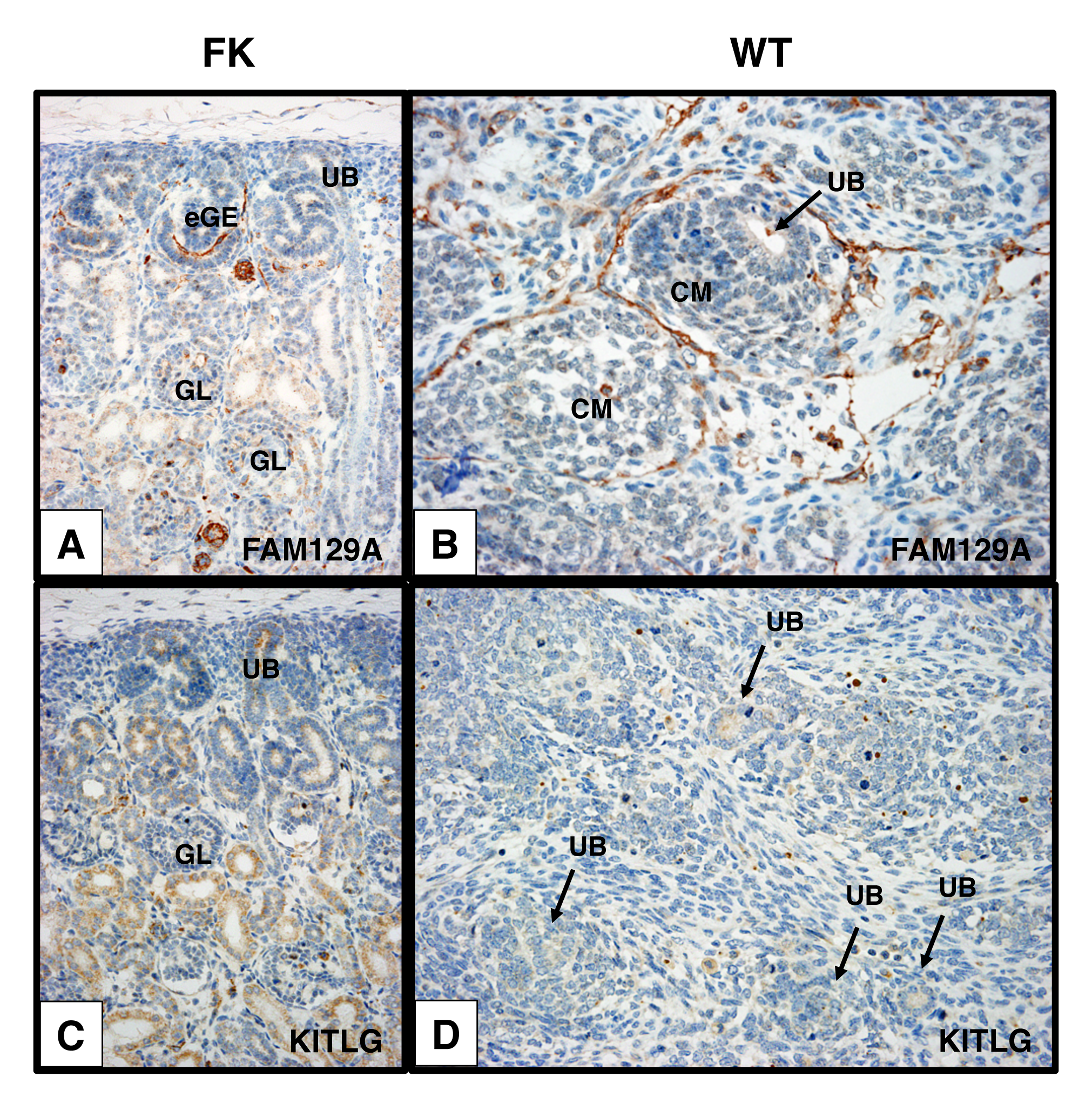

Supplement: S1 Fig — IHC for FAM129A (A, B) and KITLG (C, D) in FK and WT. (A, B) Expression of FAM129A highlights vessels in FK (A) and WT (B). (C, D) FK (C): KITLG is expressed not only in the UB but also in maturing and mature renal tubules in the cortex. WT (D): Expression of KITLG is weak in the UB/CD-like structures (arrows). Original magnification, A-D, x400. Nuclear counterstain with 3, 3’-diaminobenzidine (DAB) (TIF) [file pone.0186333.s001.tif]

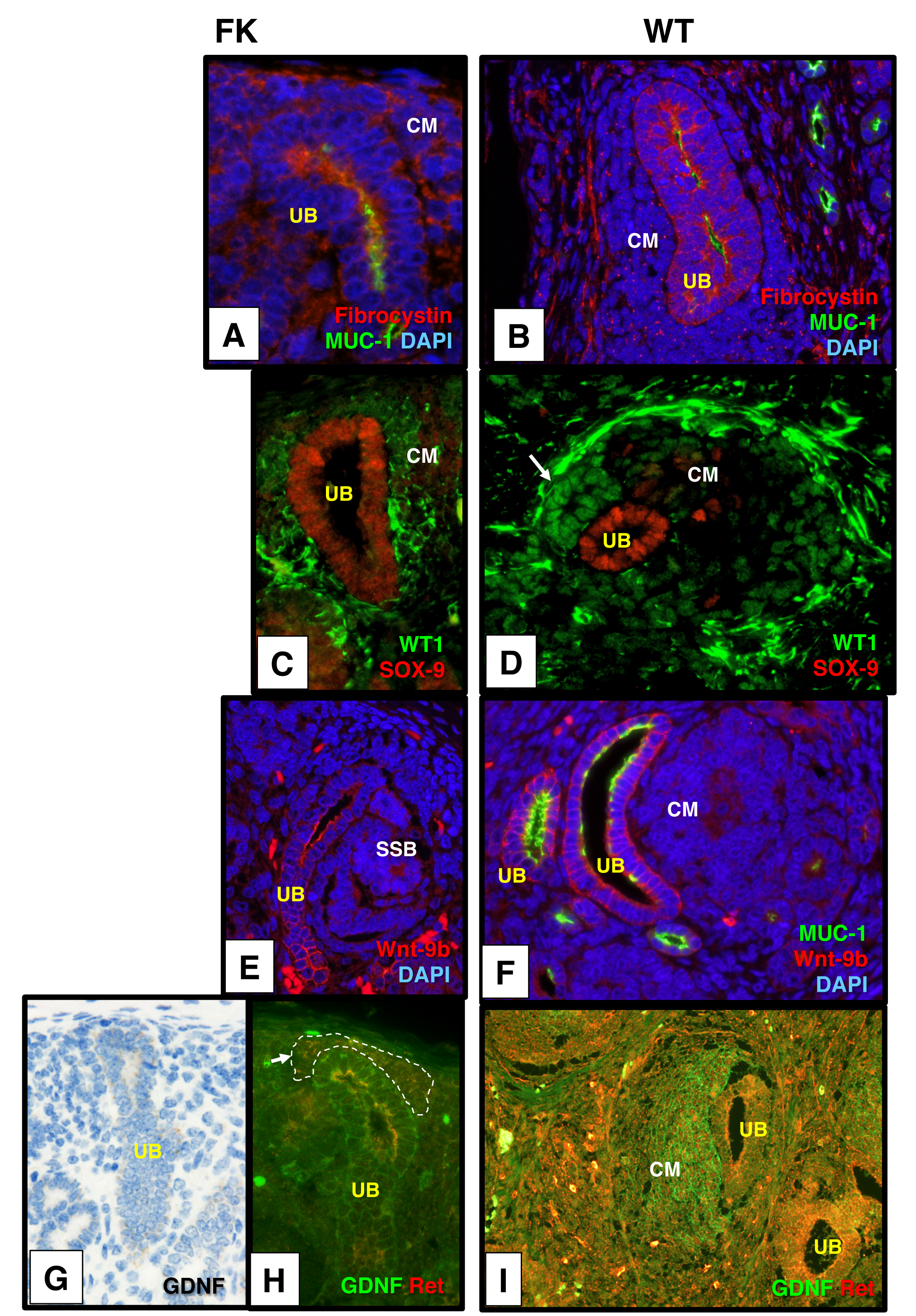

Supplement: S2 Fig — Expression of Fibrocystin (A, B), SOX9 (C, D), Wnt-9b (E, F), GDNF (G, H, I) and Ret (H, I) in FK and WT. Fibrocystin, SOX-9, Wnt-9b, and Ret did not show differential expression in fetal kidney but were expressed in the UB-like structures. (A, B) FK (A): Expression of Fibrocystin (red) in the apical membrane of a ureteric bud overlapping MUC-1expression (green). WT (B): Photomicrograph showing a central epithelial blastema pattern in which Fibrocystin (red) and MUC-1 (green) are localized to the apical membrane and cytoplasm of the UB-like structure. (C, D) Double IF for SOX-9 (red) and WT1 (green) in FK and WT. FK (C): SOX9 is expressed in the UB tip. WT (D): SOX-9 is expressed in a WT1-negative epithelium and its expression is absent in a WT1-positive epithelium (indicated by an arrow). (E, F) Double IF for Wnt-9b (red) and MUC-1 (green) in FK and WT. FK (E): Wnt-9b in the apical membrane, cell membrane, and cytoplasm of UBs. WT (F) shows an identical expression pattern in the MUC1-positive UB-like structures. (G, H, I) Expression of GDNF (green) and Ret (orange) in FK and WT. FK (G, H): GDNF is detected in and around the UB using DAB as a substrate (G). Expression of GDNF is nearly absent while that Ret is positive in the apical membrane of UB (H). Ret is also scattered in CM. WT (I): GDNF (green) and Ret (orange) are co-expressed in UB-equivalent structures and the surrounding condensing blastemal cells (CM). Original magnification, A-F, H, x400; G, x600 I, x200. (TIF) [file pone.0186333.s002.tif]

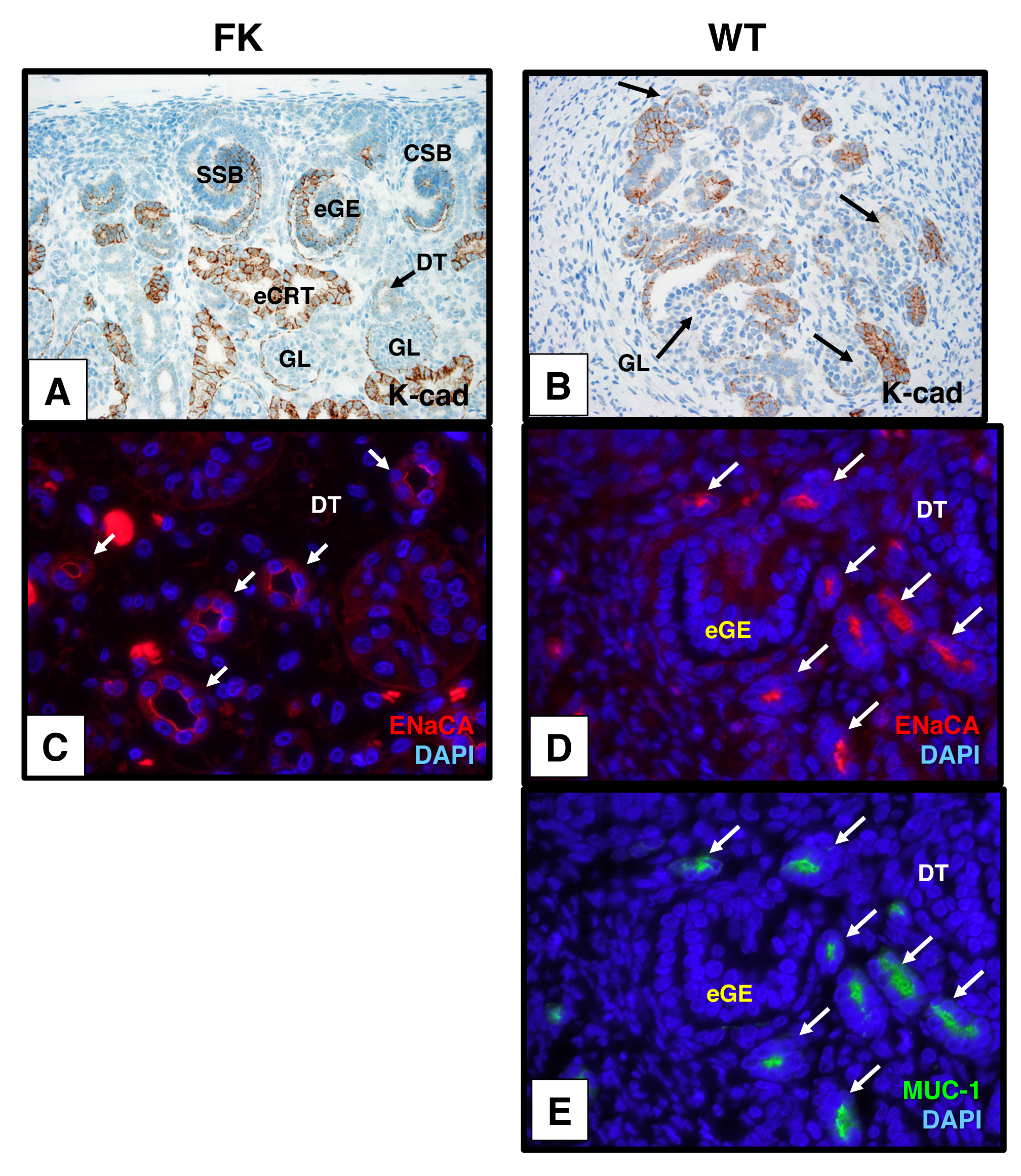

Supplement: S3 Fig — Expression of DT-related proteins [K-cadherin (A, B), and ENaCA (C, D, E)] in FK and WT. (A, B) FK (A): K-cadherin in the cell membrane of the CSB, SSB, and eGE. Its expression is also observed in ePTs and eDTs in the cortex. WT (B): Membranous and/or cytoplasmic K-cadherin expression in epithelial structures connected or adjacent to the GL-like structures (arrows). This indicates K-cadherin is involving in the formation of PT and DT. (C, D and E) FK (C): A distal tubule marker, ENaCA (red) in DT. WT (D, E): ENaCA (red) in the epithelial structures adjacent to eGE. Co-localization of MUC-1 (green) confirmed the formation of the DT (arrows). Original magnification, A-E, x400. Nuclear counterstain with DAB (A, B). (TIF) [file pone.0186333.s003.tif]

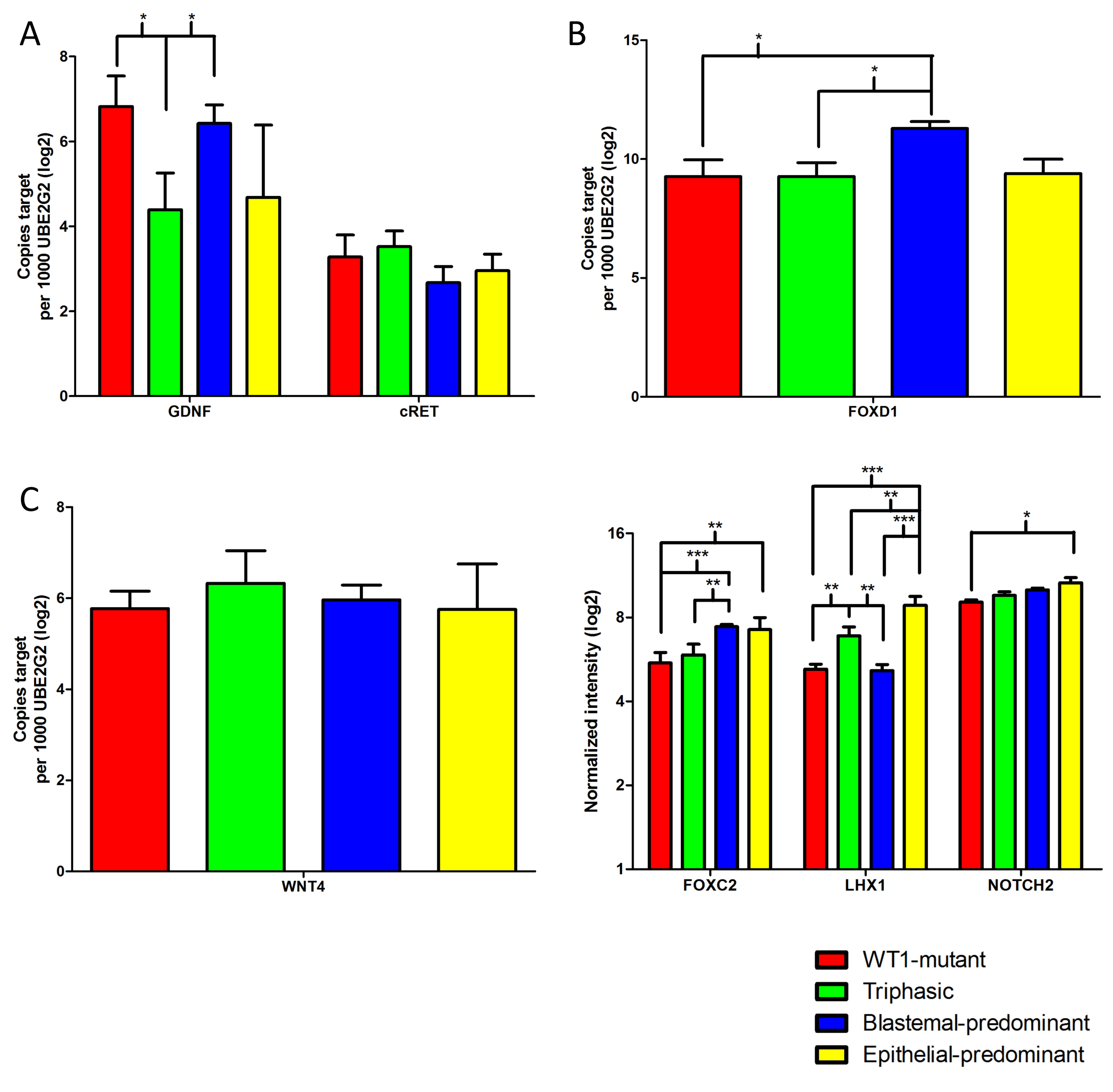

Supplement: S4 Fig — A) Expression of GDNF and cRET in WTs. GDNF and its receptor cRET are expressed in all types of WTs, with higher expression of GDNF seen in WT1-mutant and blastemal-predominant tumours. Data shown are log2-transformed QPCR expression levels normalized to UBE2G2. B) Expression of FOXD1 in WTs. Expression of FOXD1 is highest in blastemal-predominant tumours whose histology consists of non-aggregated blastemal cells with few or no UB-like structures. Data shown are log2-transformed QPCR expression levels normalized to UBE2G2. C) Expression of FOXC2, LHX1, WNT4, and NOTCH2 in WTs. FOXC2 and LHX1 are highly expressed in tumours with epithelial-predominant histology, which are predicted to be expressed in RV-equivalent structures. NOTCH2 was expressed in all histological-subtypes, with the highest expression in epithelial-predominant tumours. The graph for WNT4 shows log2-transformed QPCR expression levels normalized to UBE2G2. The second graph shows microarray log2-transformed normalized expression levels. For all graphs expression levels were compared by two-way ANOVA with Bonferroni post-tests, * = p < 0.05, ** = p < 0.01, *** = p < 0.001. (TIF) [file pone.0186333.s004.tif]

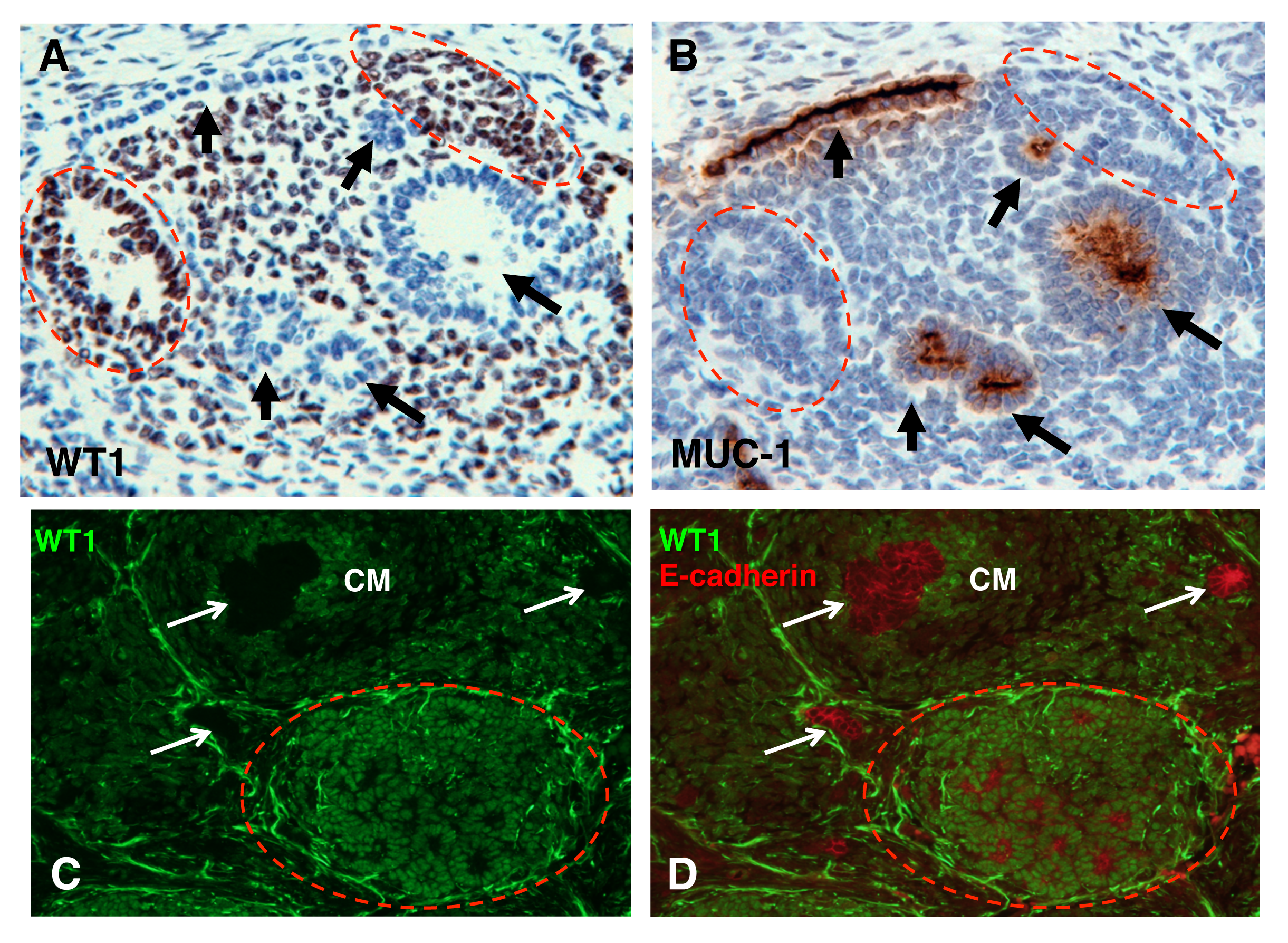

Supplement: S5 Fig — Consecutive sections show that WT1 is expressed in RV-like structures (A, surrounded by red circles) in which MUC1 expression is absent (B, indicated by arrows), in contrast, MUC1 is expressed in the UB-like structures (B) in which WT1 expression is lost (A, indicated by arrows). WT1 is also expressed in blastemal cells around the UB-like structures. WT1 (A), MUC1 (B), original magnifications: x400 (A, B). Nuclear counterstain with DAB. Nuclear immuno-positivity for WT1 highlights a cluster of RV-like cells (surrounded by a red broken circle) in a triphasic WT (C, D), while nuclear WT1 expression is absent in UB-like structures (C). E-cadherin immunostaining reveals its presence of UB-like structures surrounded by condensing mesenchyme (CM) (D). WT1 (C), WT1 and E-cad (D), original magnifications: x200 (C, D). (TIF) [file pone.0186333.s005.tif]

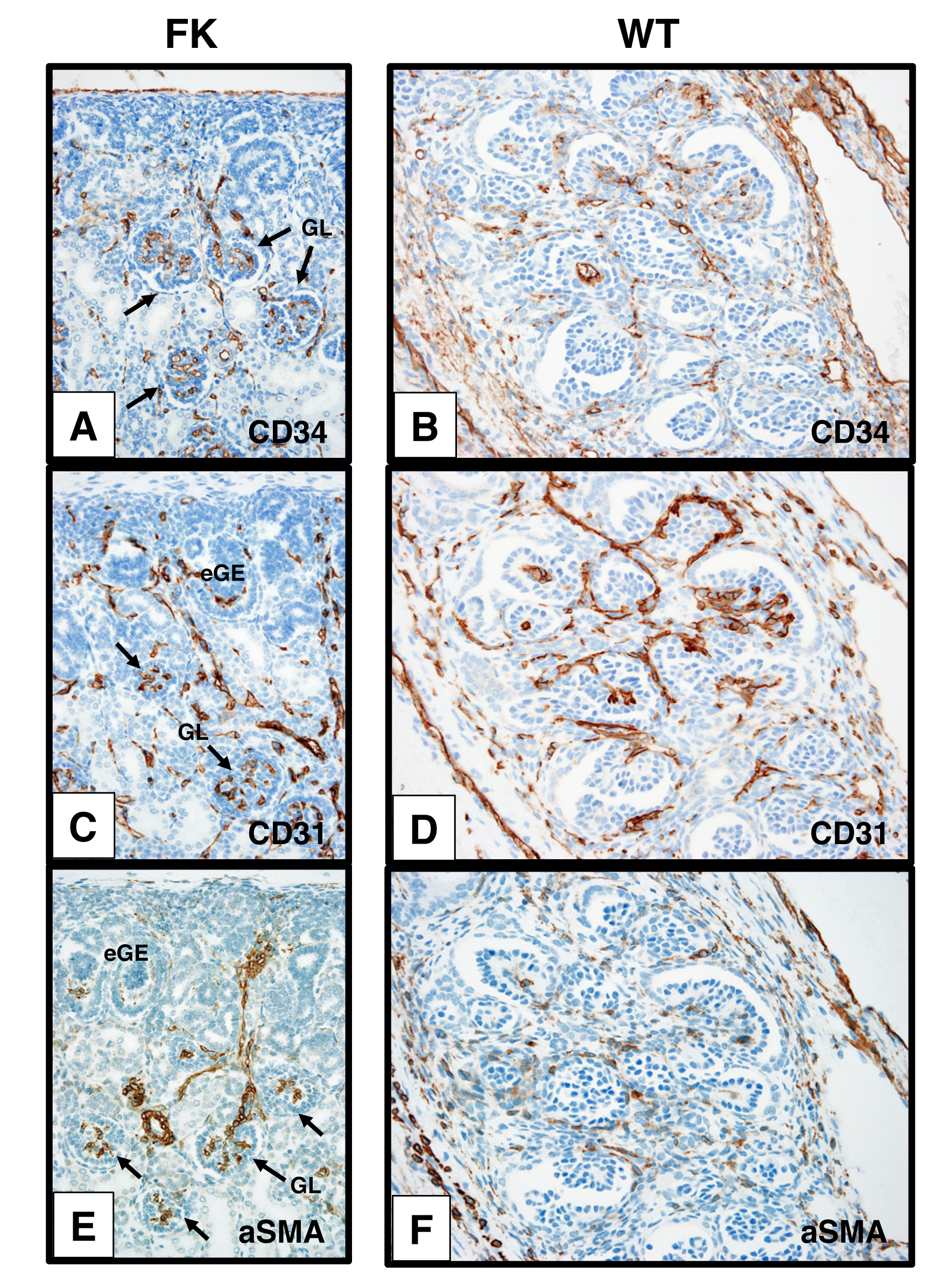

Supplement: S6 Fig — The vasculization phase [CD34 (A, B), CD31 (C, D), alpha SMA (E, F)] in FK and WT. FK: CD34 (A), CD31 (C), and alpha SMA (D) revealing vasculogenesis in eGE and G (arrows). WT: CD34 (B), CD31 (D), and alpha SMA (F), An area of the formation of GL-like structures showing vasculogenesis. Original magnification, A-F, x400. Nuclear counterstain with DAB. (TIF) [file pone.0186333.s006.tif]
